# Supplementary material for: Human cone photoreceptor transplantation stimulates remodeling and restores function in AIPL1 model of end-stage Leber congenital amaurosis
Source: Stem Cell Reports. 2025 Mar 27;20(4):102470. doi: 10.1016/j.stemcr.2025.102470 (PMC12069896; doi:10.1016/j.stemcr.2025.102470)
Supplement: Document S1. Figures S1–S7 and Table S1 [file mmc1.pdf]

Stem Cell Reports, Volume *20*

Supplemental Information

Human cone photoreceptor transplantation stimulates remodeling and restores function in AIPL1 model of end-stage Leber congenital amaurosis

Christopher A. Procyk, Anna Melati, Joana Ribeiro, Jingshu Liu, Matthew J. Branch, Jamie D. Delicata, Menahil Tariq, Aikaterini A. Kalarygrou, Jessica Kapadia, Majid Moshtagh Khorsani, Emma L. West, Alexander J. Smith, Anai Gonzalez-Cordero, Robin R. Ali, and Rachael A. Pearson

SUPPLEMENTAL INFORMATION

Contains: Supplemental Figures S1 – S7 and figure legends

Supplemental Experimental Methods

SUPPLEMENTAL FIGURES

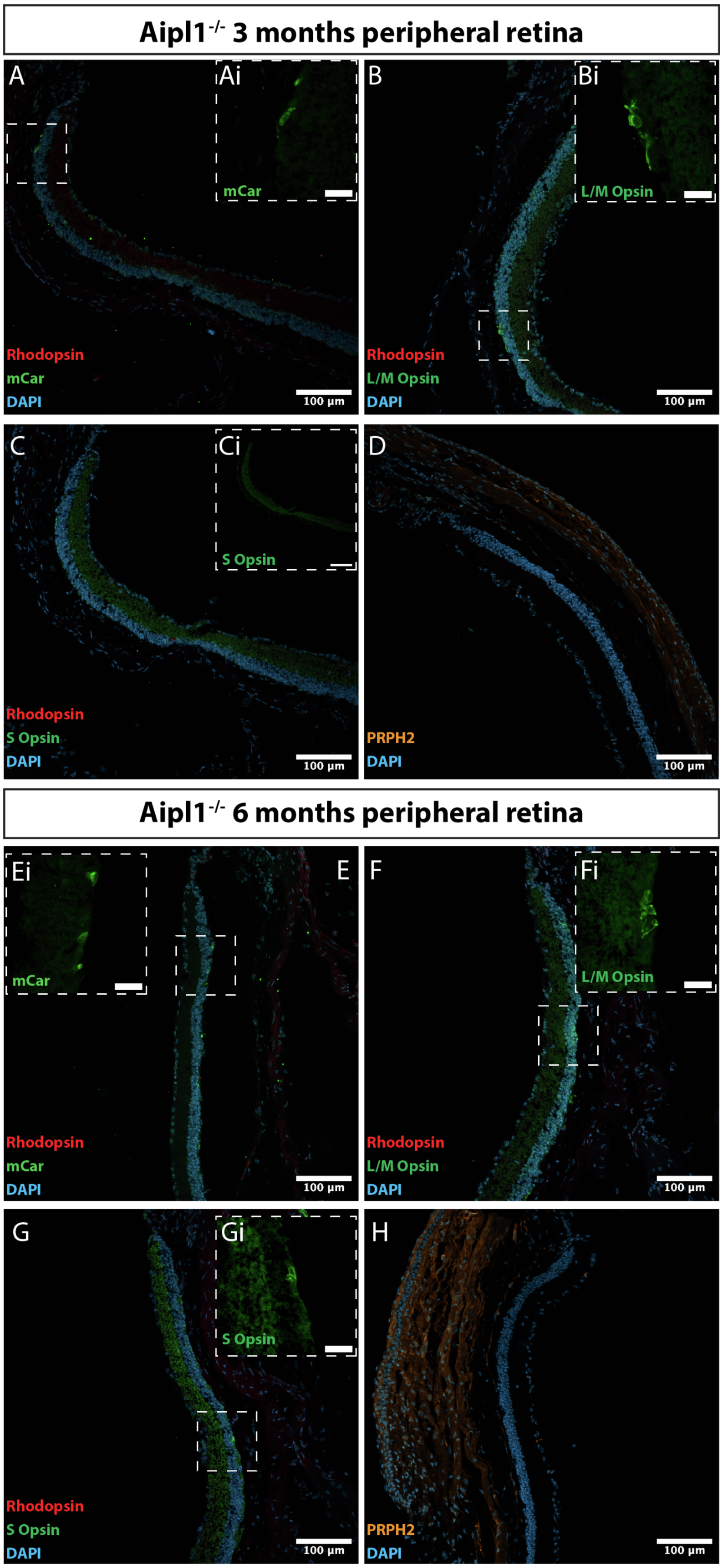

Supplemental Figure S1. Photoreceptor degeneration in the periphery of *Aipl1$^{-/-}$* retina

(A-D) Periphery of representative 3-month-old *Aipl1$^{-/-}$* retinae immunolabelled for rod (Rhodopsin, *red*) and cone (mCar, M/L opsin, S Opsin, *green*) photoreceptor markers. No rhodopsin+ rods were seen at 3 months of age in the *Aipl1$^{-/-}$* peripheral retina. Occasional mCar+ and L/M opsin+ cones were seen, but S opsin was not detected **(Ai, Bi, Ci)**. No labelling for the outer segment protein Prph2+ was seen **(D)**. **(E-H)** At 6 months of age the ONL of *Aipl1$^{-/-}$* mice is completely degenerated, with only very sporadic cones seen in the periphery **(Ei, Fi, Gi)**. No Prph2+ labelling was visible **(H)**. Scale bar: A-H, 50μm; Ai, Bi, Ci, Ei, Fi, Gi, 20μm.

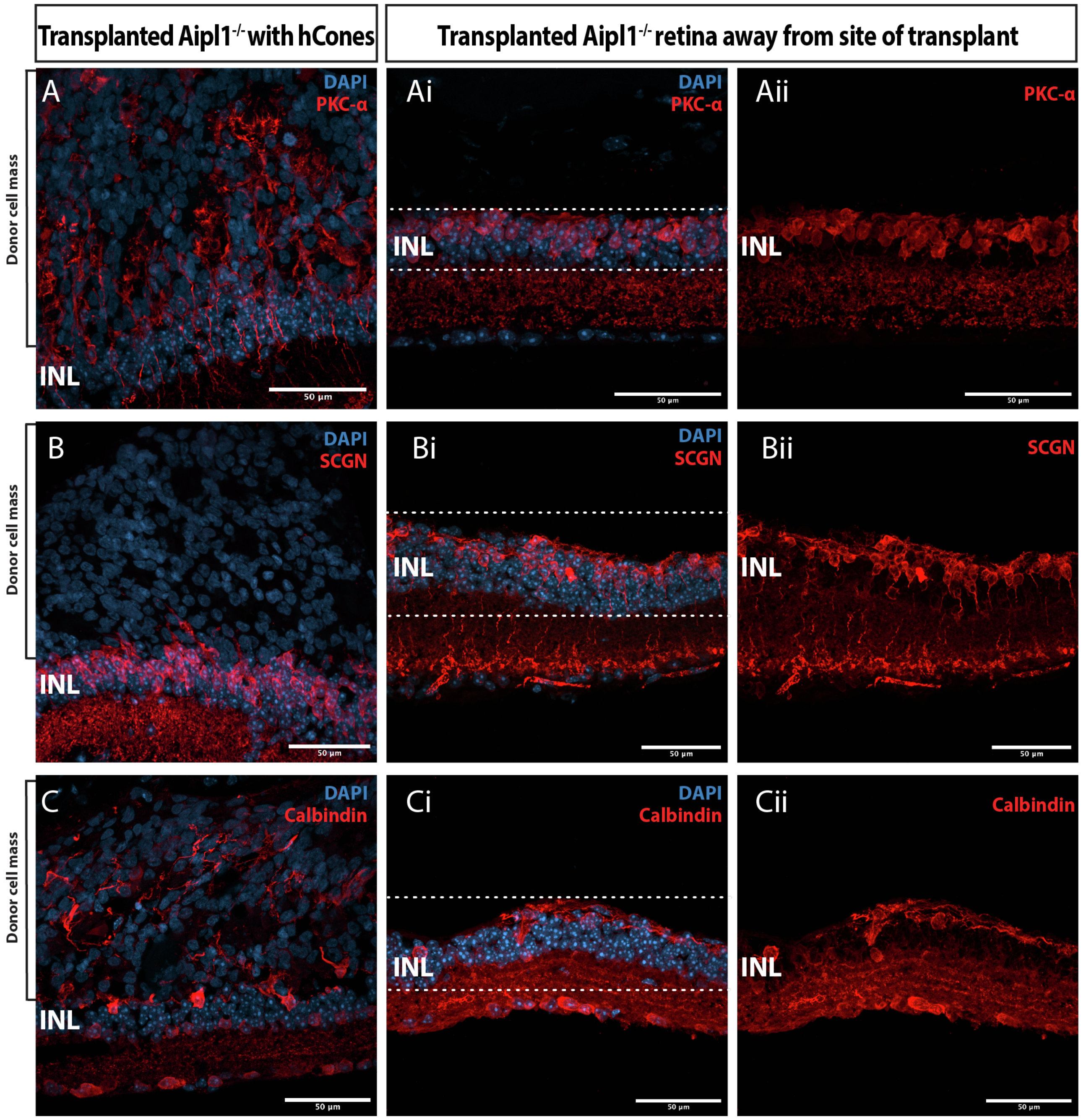

Supplemental Figure S2: INL undergoes extensive remodelling in regions immediately beneath transplanted with human cones. (A-C) Different regions in the same retinae with or without hCone donor cell mass show markedly different extents of neural remodelling. **(A-Aii)** PKC-α+ ON BCs show significant dendritic extension up into the hCone donor cell mass, while in the same retina away from the engraftment site, BCs show no neurite remodelling. **(B-Bii)** SCGN staining for cone BCs shows a similar pattern. **(B)** cone BCs show clear neurite extension toward the donor cell mass, while cone BCs located distant to the donor cell mass extend their dendrites horizontally, similar to age-matched

Aipl1$^{-/-}$ degenerated retina. **(C-Cii)** HCs, immunolabelled for calbindin, exhibit the same pattern of remodelling, those under the donor cell mass extending neurites towards it, while those distant to the donor cell mass remain unchanged from control *Aipl1$^{-/-}$*. Scale bar 50μm. INL – inner nuclear layer. Dapi – nuclear label.

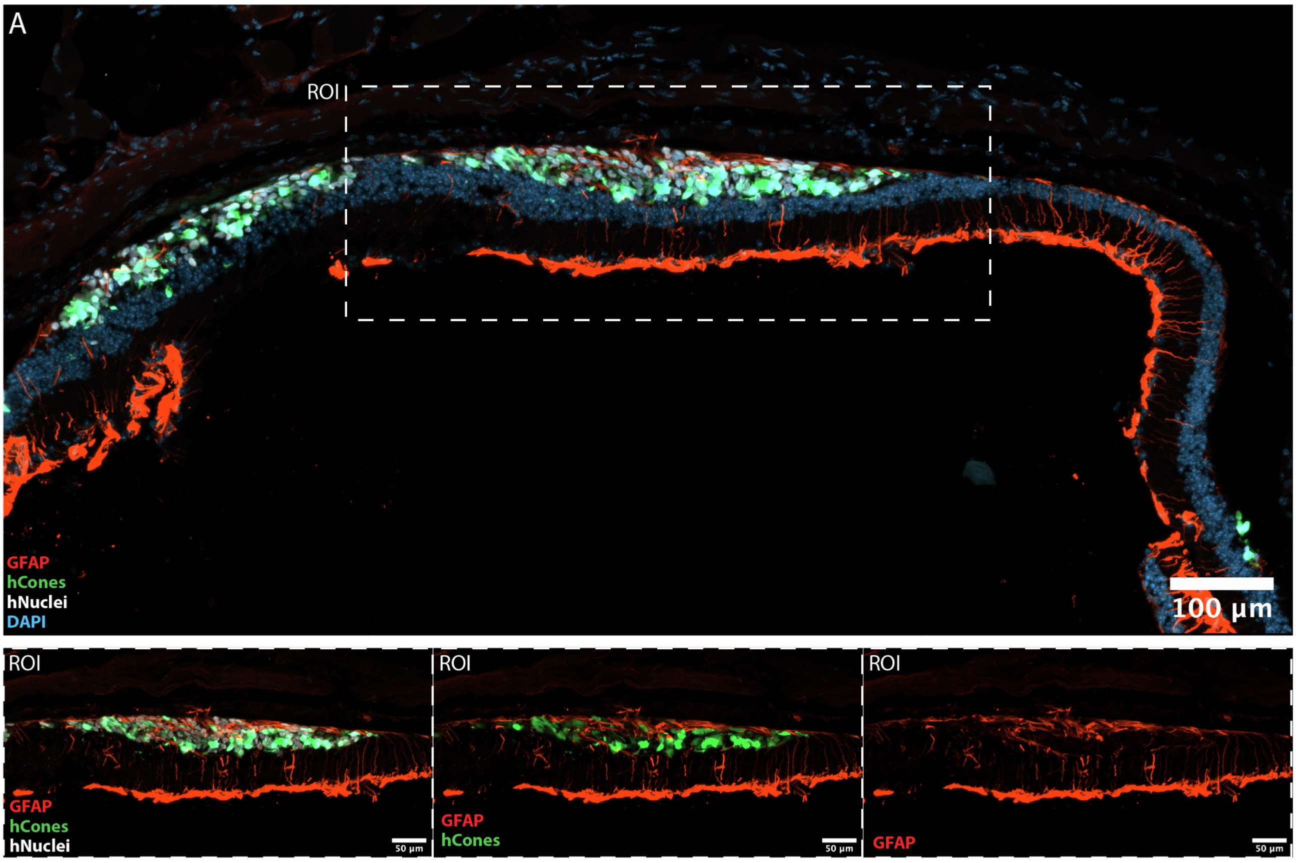

Supplemental Figure S3: Muller glia cells within the recipient retina extend into and delineate the outer edge of the human cone graft. (A) In regions of the retina with GFP+ hCones (*green*), Müller glial cells are activated, and upregulate Gfap (*red*). The processes of Müller glia cells extend from the ganglion cell layer through the host retina towards the hCone donor cell mass. HNA (hNuclei) colocalises with GFP+ hCones but not Gfap+ Müller glia, indicating that the Müller glia predominantly derive from the host mouse retina. **ROIs** show glial cell processes extending up through the graft, delineating its apical margin thereby incorporating the graft within the host retinal structure. Main image is a confocal maximum image projection tile scan (digital stitching), ROIs are digital zooms from the same tile scan. Scale bar 100 µm, 50 µm for ROIs.

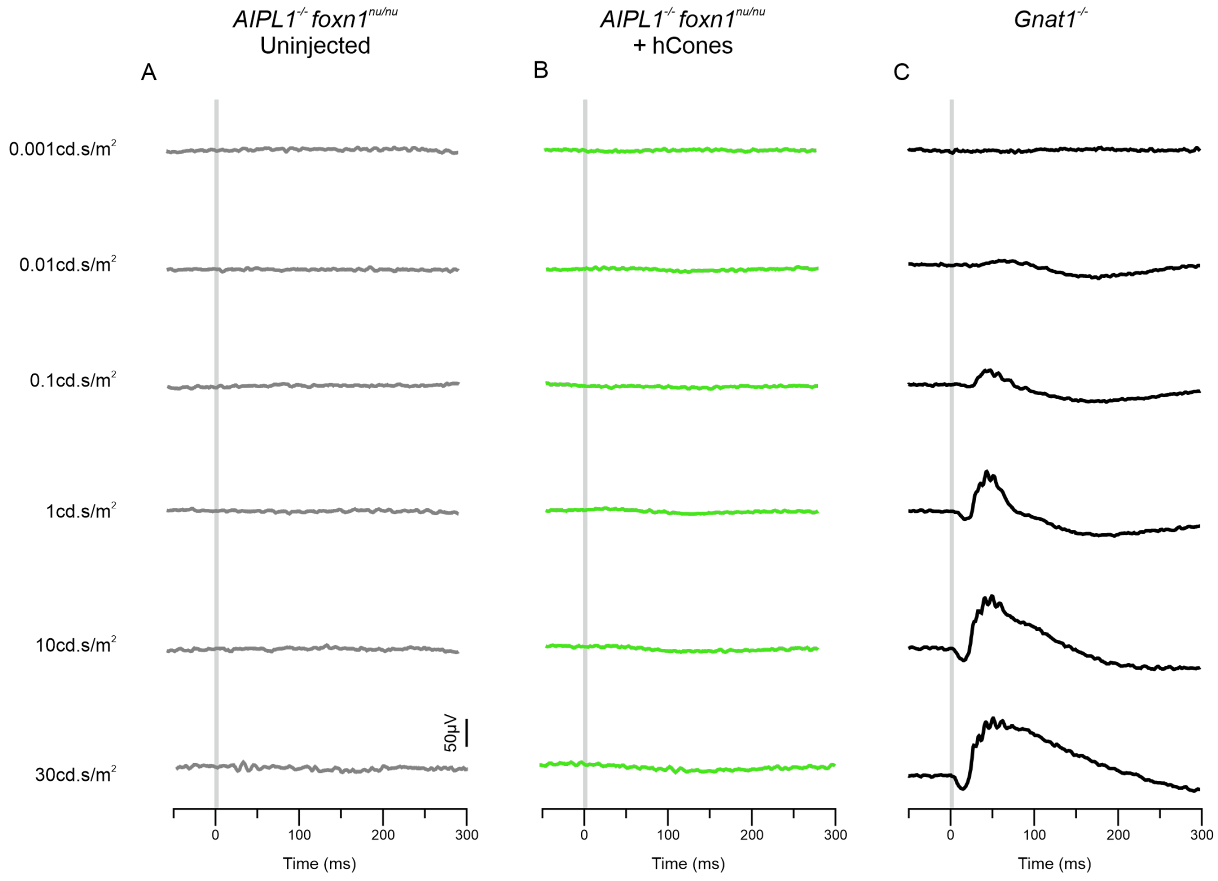

Supplemental Figure S4: Transplanted human cones do not drive reproducible ERG responses in the *Aipl1*$^{-/-}$ + human cones treated retina

A-C, Mean dark-adapted ERG traces from all animals recorded at 6 months age in (**A**) uninjected *Aipl1*$^{-/-}$ mice (N = 3), **(B)** *Aipl1*$^{-/-}$ + hCone transplanted mice, 3 months post-transplantation (N = 4) and **(C),** age-matched *Gnat1*$^{-/-}$ mice (N = 4), which exhibit cone-only mediated function and serve as a positive control. A small response was observed in *Gnat1*$^{-/-}$ animals from 0.01 cd.s/m^2, with measurable a- and b-waves from 0.1 cd.s/m^2 but no reproducible response was seen in either the un-injected or the transplanted *Aipl1*$^{-/-}$ mice at any intensity.

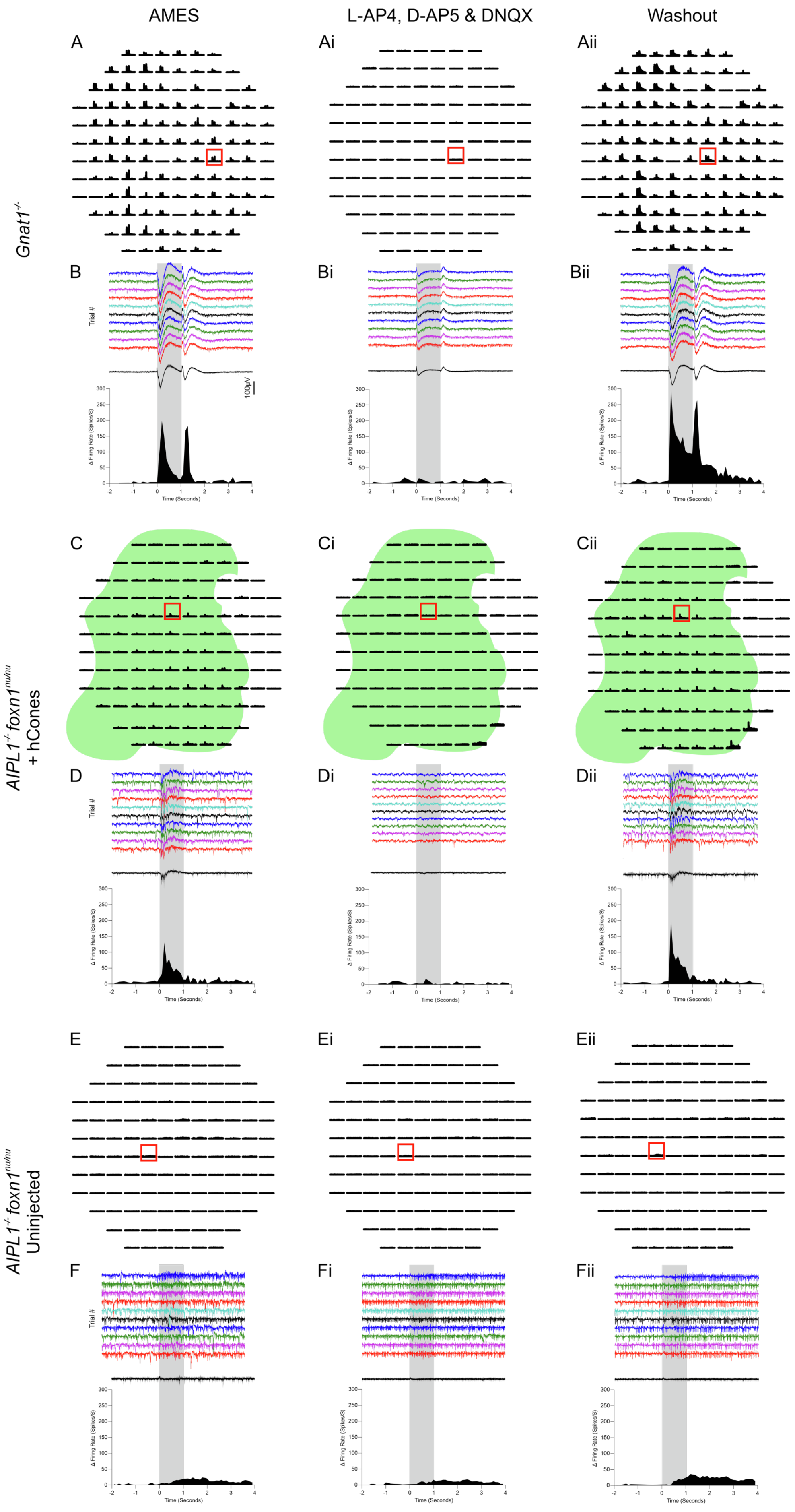

AMES
L-AP4, D-AP5 & DNQX
Washout
A
Ai
Aii
Gnat1-/-
B
Bi
Bii
Trial #
100µV
Δ Firing Rate (Spikes/S)
Time (Seconds)
C
Ci
Cii
AIPL1-/- foxn1nu/nu + hCones
D
Di
Dii
E
Ei
Eii
AIPL1-/- foxn1nu/nu Uninjected
F
Fi
Fii

Supplemental Figure S5: Transplanted human cones connect to the host *Aipl1*$^{-/-}$ retina through functional glutamatergic synapses in the outer retina

Multi-unit spiking activity before, during, and after synaptic blockade. (**A**) Representative *Gnat1*$^{-/-}$ retina. Transient increases and decreases in firing rate were observed following light onset and/or offset. (**Ai & Aii)** These were reversibly abolished by synaptic blockers. (**B-Bii**) mERG and multi-unit PSTH (magnified from the *red box* in **A**) shows fast cone-driven response, which is reversibly abolished by pharmacological intervention. (**C**) Representative *Aipl1*$^{-/-}$+ hCone transplanted retina. Transient increases in firing rate correlated with the position of overlying GFP+ hCones following stimulation with a 1s light pulse. (**Ci & Cii)** Addition of synaptic blockers reversibly abolished these responses. (**D-Dii**) Light-evoked mERG and multi-unit PSTH (*red box* in **C**) illustrates hCone-driven light responses that are both abolished by pharmacological intervention and return following washout. (**E**) Representative untreated age-matched *Aipl1*$^{-/-}$ retina. Most channels were not light responsive and (**Ei & Eii)** there was no effect of synaptic blockers on these few light responsive channels. (**F-Fii**) No discernible mERGs were seen on the few channels which demonstrated a deafferented ipRGC responses following light onset (*red box*), which were not eradicated by synaptic blockers, as expected for deafferented intrinsically photosensitive retinal ganglion cell responses.[30]

Scale bars: 400 Spikes/s, 5 s (A, C, and E); Green overlay indicates region of GFP+ cell mass. Grey bars in B, D & F indicate duration of light pulse.

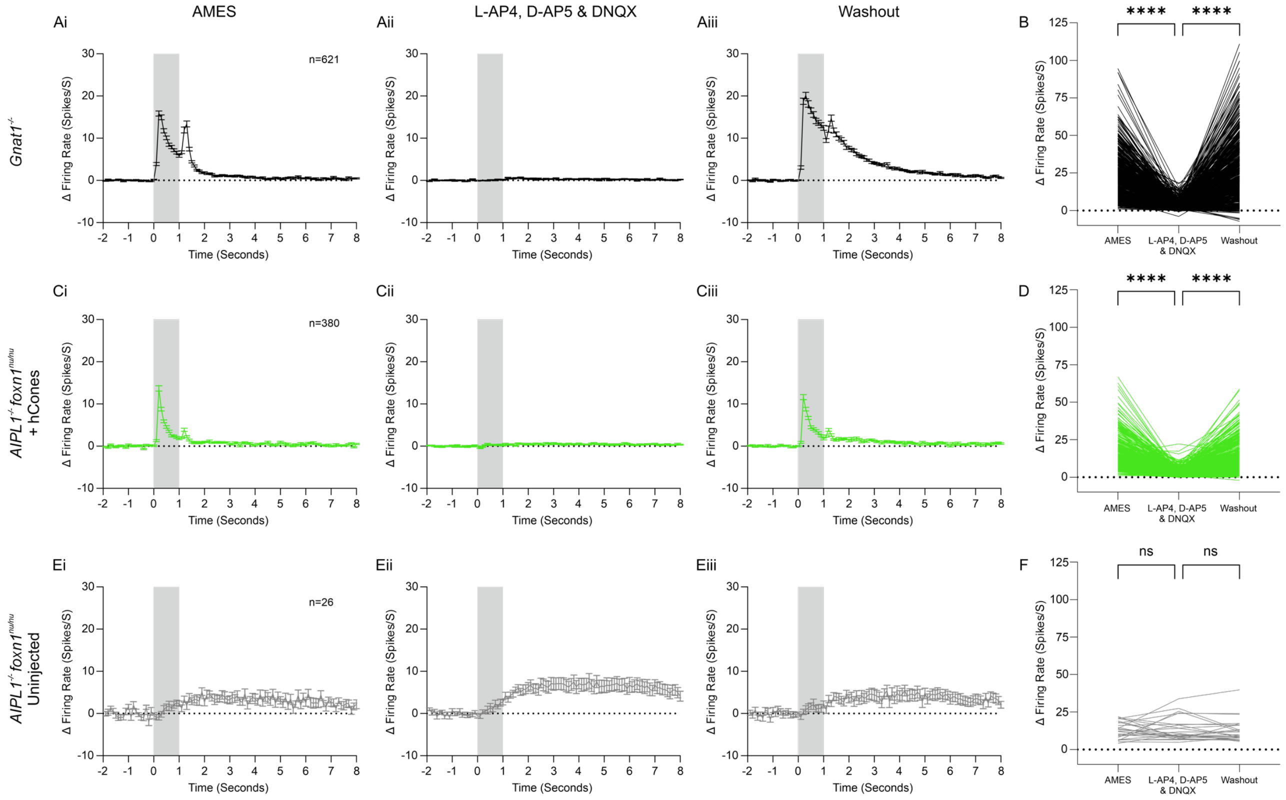

Supplemental Figure S6: Single unit quantification of Glutamatergic transmission of visual information at the human cone-host bipolar cell synapse.

(**A**) Average PSTH (Mean ±SEM) of all ON-type responses in *Gnat1*$^{-/-}$ retinas (n=621 units) demonstrates a robust light response when stimulated with a 1s light pulse from darkness (*left*), which is eradicated during application of synaptic blockers (middle) and returns following Washout (*right*). (**B**) Peak ON response amplitude of all single units in A demonstrates a significant reduction in amplitude under synaptic blockade (Mean ±SEM; 2.75 ± 0.13 Spikes/s) when compared to the AMES (22.42 ± 0.6 Spikes/s) and Washout conditions (25.95 ± 0.84 Spikes/s; 2-Way Anova; $p < 0.001$). (**C**) Average PSTH of ON-type responses in *Aipl1*$^{-/-}$ + human cone transplanted retinas (n = 380) show a robust light response to the same 1s light pulse from darkness, (*left*) which is eradicated during synaptic blockade (middle) and returns upon Washout (*right*). (**D**) Peak ON response Amplitude of all single units in C demonstrates a significant reduction in amplitude under synaptic blockade (3.21± 0.14 Spikes/s) when compared to the AMES (16.79 ± 0.56 Spikes/s) and Washout conditions (14.95 ± 0.56 Spikes/s; 2-Way Anova; $p < 0.001$). **(E)** Average PSTH of all ON-type responses in untreated *Aipl1*$^{-/-}$ retinas (n= 26 units) demonstrates slow and sustained light responses originated from intrinsically photosensitive retinal ganglion cells

when stimulated with a 1s light pulse from darkness (*left*), which is not eradicated during application of synaptic blockers (*middle*) and remains following Washout (*right*). **(F)** Peak response amplitude is not significantly different under synaptic blockade (13.32 ± 1.56 Spikes/s) when compared to AMES (12.63 ± 1.06 Spikes/s; 2 Way Anova, $p = 0.85$) or Washout conditions (12.38 ± 1.53 Spikes/s; 2-Way Anova; $p = 0.74$)

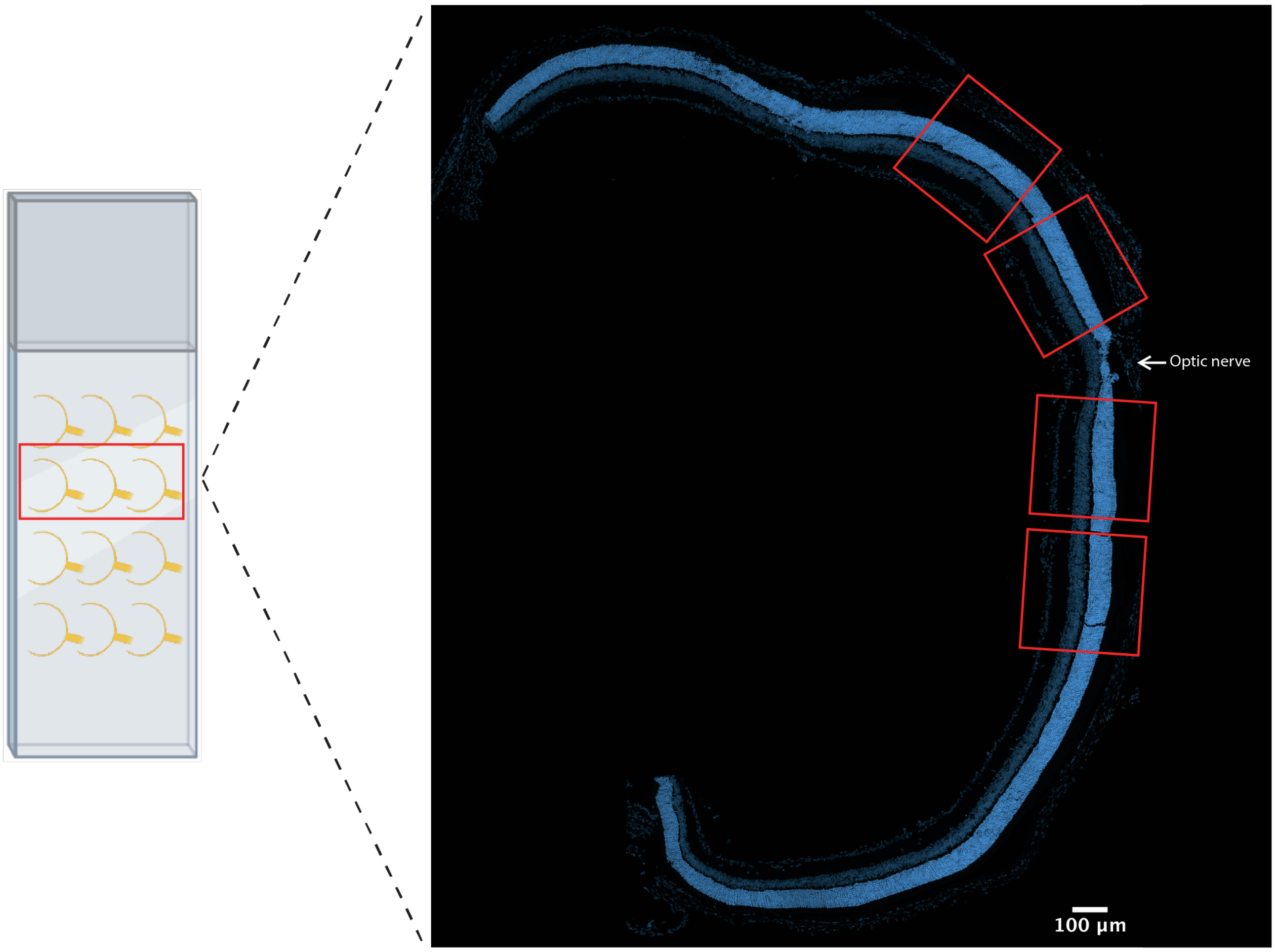

Supplemental Figure S7. Schematic and confocal tile scan (digital stitching) image showing the locations of the four regions (*red boxes*) selected for imaging of immunohistological assessments of inner retinal remodelling.

SUPPLEMENTARY EXPERIMENTAL METHODS

EXPERIMENTAL PROCEDURES

Animals

Aipl1$^{-/-}$, *Aipl1*$^{-/-}$/*FoxN1*$^{nu/nu}$ (line generated in-house), *Gnat1*$^{-/-}$ (Calvert *et al*., 2000), and *C57Bl/6* (Charles River) animals were maintained on a standard 12 hr light-dark cycle. Mice received food and water *ad libitum* and were provided with fresh bedding and nesting daily. Male and female mice were used without discrimination for experiments and are represented in approximately equal numbers. All experiments have been conducted in accordance with the United Kingdom Animals (Scientific Procedure) Act of 1986 and Policies on the Use of Animals and Humans in Neuroscience Research and ARVO Statement for the Use of Animals in Ophthalmic and Vision Research.

hESC maintenance culture

The human H9 ESC line (WA09, female, ID/registry: WAe009-A (hPSCreg); Lot RB66492, P30) was acquired directly from WiCell and used in accordance with the ISSCR Standards for Human Stem Cell Use in Research. In brief, a working cell bank (WCB) was cryopreserved at P33 in Knockout™ Serum Replacement (#10828010, ThermoFisher) with 10% CryoSure DMSO (#WAK-DMSO-10, WAK). These cells were fully characterised, including Karyometrix analysis (Stemnovate) with no major chromosomal changes detected (>60bps). The undifferentiated state of the cell population was assessed by flow cytometry (Human Pluripotent SC Analysis kit, #560461 and #560589, BD Biosciences) with all pluripotent markers present in >70% of cells and differentiation markers present in <10% of cells. Each experimental run initiated from a freshly thawed vial of the WCB, with cells seeded for differentiation within 15 passages. ESCs were maintained under feeder-free conditions in Essential 8™ media (#A1517001; ThermoFisher) on Geltrex™ (#A1413301;

ThermoFisher) coated 6 well plates. They were fed daily and grown to between 60-80% confluence, before passaging with Versene (#15040066; ThermoFisher) and seeded at a density of $2x10^4$ cells/cm^2. Routine mycoplasma (MycoAlert plus detection kit, #LT07-710, Lonza) and sterility testing (Tryptic Soy broth, #1463170010 and Fluid Thioglycollate media, #STBMFTM12, Merck) was performed monthly for all cell cultures.

Retinal differentiation culture and photoreceptor transplantation

ESCs were differentiated into retinal organoids (hROs), transduced with *ShH10.2.1L/MOpsin.GFP* virus, and GFP^{+ve} human cones were isolated at 17-21 wks of differentiation, as we have previously described (Gonzalez-Cordero et al., 2017; Ribeiro et al., 2021) with minor modifications. GFP+ purity was > 80% in all transplants (mean 81.0% ± 12.3, N = 4 sorts), as verified by flow cytometry post-sort. Sorted cells were re-suspended in EBSS at a concentration of 5×10^5 / 2μL and administered by subretinal injection. All physiological and histological assessments were performed between 10-14 weeks (~3 months) post-transplantation.

Surgery and transplantation

All surfaces were wiped with 70% ethanol prior to surgery. Mice were anesthetized by intraperitoneal injection (10 mL/Kg) of a mixture of a mixture of Dormitor (1 mg/ml medetomidine hydrochloride; Pfizer Pharmaceuticals), ketamine (100 mg/ml; Fort Dodge Animal Health), and sterile water in the ratio 5:3:42. Tropicamide (1 %; Bausch & Lomb) was used to dilate the pupils and topical anaesthetic was applied (Tetracaine). Eyes were kept moist by using Viscotears (Novartis Pharmaceuticals UK Ltd). Surgery was performed under direct visual control using an operating microscope (Zeiss). A sterile 34-gauge hypodermic needle was used to make a transcleral entry in the posterior orbit and slowly inject 2 μl of

cell suspension into the sub-retinal space. The same region of the eye was targeted for all injections. Significant care was taken not to rupture the very thin remaining neural retina. Anaesthesia was then reversed using same amount of Antisedan, 10 mL/Kg, (atipamezole hydrochloride 0.10 mg/ml, Pfizer Pharmaceuticals, Kent UK), with the mice placed on heat mats until fully recovered.

Histology and immunohistochemistry

Mice were euthanized by cervical dislocation 3 months post-transplantation, unless otherwise noted. Eyes were dissected to remove the cornea, iris, and lens. All samples were fixed in 4% PFA, cryopreserved in 20% sucrose (30 minutes at room temperature or overnight at 4°C) and embedded in OCT before samples were cryo-sectioned at 12 μm thickness across 6 slides and stored at –20°C. Immunohistochemistry was performed broadly as previously described (Ribeiro et al., 2021), with some modifications. A list of primary and secondary antibodies and staining conditions is provided in **Supplemental Table S1**. Samples were imaged using a Zeiss LSM900 confocal microscope. Images shown are maximum projection images (MIPs) of xyz stacks, at 1μm intervals in the z plane, unless otherwise stated (whereupon single section images may be shown to improve clarity and/or confirm co-localization). Zeiss LSM image software, Imaris, ImageJ and Adobe Photoshop were used for image processing.

Cell quantification

To quantify PKC-α, Secretagogin and HuC/D positive cells, confocal images (162.5 x 162.5 μm) were taken of x4 Regions of Interest (ROI) from the superior mid/central retina, from 3 sections per retina from the region containing the optic nerve (**Supplementary Figure S7**). Counts were made at 3 months and 6 months of age, or as detailed in the Results. All counts

included age matched wildtype (*C57Bl/6J*) controls and were performed by a researcher that was masked to the age and strain of the animals.

To quantify nuclear size and HNA expression, eyes bearing comparable-sized cell masses were used. Nuclear size was determined by selecting cells at random and measuring along the longest axis using ImageJ. Nuclei size in mouse cones were measured using the *Chrnb4.eGFP* cone reporter line. Human nuclei measurements were taken from 2.1L/MOPSIN.GFP^{+ve}/HNA^{+ve} cones within the donor cell. At least N = 3 retinas were used for each group.

Retinal and Visual Function tests

Optomotor response

Ten weeks after human cone transplantation, the optokinetic reflex of recipient mice was assessed using the OptoDrum system (Striatech, Tübingen, Germany). Mice were kept under ambient light conditions and pupil dilation was not performed. Briefly, mice were placed on an elevated central platform surrounded by four computer screens displaying a striped pattern rotating at 12 degree/second. By changing contrast and spatial frequency of the stripes while monitoring the reflexive head movement of the test mouse, the threshold of its vision was determined. The visual acuity was tested at 99.72% contrast and the contrast sensitivity was measured at 0.122 cycle/degree. Note that the maximum irradiance emitted by the screens as measured at the platform was $8.8x10^{13}$ photons cm^2/s. The reduced sensitivity of Gnat1$^{-/-}$ (cone-only) retinas, compared to wildtype mice, indicates that it is unlikely to maximally stimulate human cones. The optokinetic response was automatically detected and analysed by the OptoDrum software in an unbiased manner. The left and right eyes were tested separately by changing the rotating direction of the visual stimulus (clockwise for the left eye and counterclockwise for the right eye). Each eye was

tested on 3 separate occasions within one week. *Gnat1*$^{-/-}$ and *C57Bl/6* wildtype mice served as positive controls for cone-only and rod/cone function. All individual test outcomes from all animals assessed are shown. N = 15 *Aipl1*$^{-/-}$ treated eyes, N = 9 *Aipl1*$^{-/-}$ untreated eyes, N = 6 *Gnat1*$^{-/-}$ eyes and N = 6 *C57Bl/6* eyes were tested. Note that x3 *Aipl1*$^{-/-}$ received no treatment, the remainder had one or both eyes treated.

Electroretinogram recordings (ERGs)

Four *Gnat1*$^{-/-}$, four *Aipl1*$^{-/-}$ hCone transplanted mice and four untreated *Aipl1*$^{-/-}$ mice underwent dark adapted ERG recording using a Celeris ERG system with a full field stimulator (Diagnosys). A masked protocol was employed such that the person performing the ERGs and analysis did not know which eyes received treatment. Animals were dark adapted for a minimum of 6 hours prior to ERG before being anaesthetized as above and pupils dilated using a combination of Tropicamide (1%) and phenylephrine (2.5%). Viscotears were placed on each cornea to keep them moistened and facilitate contact with corneal electrodes. Animals were kept warm throughout recording. The ERG protocol was run in the “Touch/Touch” modality, in which eyes are recorded sequentially with the non-recorded eye acting as the reference electrode. Dark adapted, single flash recordings were obtained at light intensities of 0.001, 0.01, 0.1, 1, 10 and 30 cd.s/m2 with a flash duration of 4ms and interstimulus intervals of 5 s, 5 s, 5 s, 10 s, 30 s, 30 s respectively. Data were recorded from 50 ms before stimulus onset to 300 ms post-stimulus with a sampling frequency of 2 kHz. Ten responses were averaged for light intensities of 0.001, 0.01, 0.1, and 1 cd.s/m2 and 5 responses for 10 and 30 cd.s/m2. The bandpass filter was set between 0.125 and 300 Hz.

For analysis, the a- and b-wave amplitudes (a-wave trough to b-wave peak) were measured. The mean time to peak for *Gnat1*$^{-/-}$ mice (N = 4) was determined for each intensity and this time point used to determine a- and b- wave amplitudes in the other lines, if not clear.

MEA

Gnat1$^{-/-}$ (N = 4), human cone transplanted *Aipl1*$^{-/-}$ (N = 4) and untreated *Aipl1*$^{-/-}$ (N = 3) mice were euthanized by cervical dislocation ~3 months post-transplantation. Eyes were immediately enucleated, and retinal isolation was performed in the dark in warm carboxygenated (95% O2-5% CO2) AMES media supplemented with 1.9 g/L sodium bicarbonate (Sigma Aldrich, UK). The retina was incised four times in a Maltese cross motif and mounted onto a perforated Multi Electrode Array (120pMEA100/30iR-ITO; MultiChannel Systems, Reutlingen, Germany) with the ganglion cell layer facing down onto the electrodes. For transplanted animals, GFP expressing regions of the retinal cell mass were placed centrally over the electrodes to maximize the recording area covered by transplanted human cones. A platinum harp (0.8g) bearing a framework of parallel silicon strings was used to hold the retina in place and keep it stable during recording. The MEA chamber was then mounted into the head stage (MEA2100-120 head stage; Multi Channel Systems). Electrophysiological signals were digitised and recorded with a sampling frequency of 20 kHz using Multi Channel Experimenter (Multi Channel Systems). Prior to recording, the retina was allowed to rest for 30 minutes of dark adaptation and to allow for neuronal activity to stabilize. To preserve physiological conditions, the tissue was perfused with carboxygenated AMES (PPS2; Multi Channel Systems) and 9-cis-retinaldehyde (Sigma Aldrich; UK) and maintained at 36°C (TC02 controller; Multi Channel Systems) throughout the duration of the experiment.

Presentation of visual stimuli

Light stimuli were designed using MC_Stimulus II (Multi Channel systems), which programmed a T-Cube LED Driver (Thorlabs; Germany) to control a mounted Cyan LED (λmax = 505nm; M505L4; Thorlabs). A light guide was used to project light from the LED onto the retina from above (Maximum irradiance at retinal surface = 1.12×10^{15} photons cm^2/s). All light measurements were recorded using a calibrated spectroradiometer (ILT960VIS-RAA4; Pro Lite Technology)

Light stimuli & pharmacology

1s Flashes

Full-field 1 s light steps were presented from darkness with a 10 s interstimulus interval and repeated 10 times.

Sensitivity

In all animals, 10 repeats of a full-field 100 ms light steps with a 10 s interstimulus interval, were presented at nine increasing light intensities from a dark-adapted background (maximum irradiance = 1.12×10^{15} photons cm^2/s). The stimulus sequence started from the lowest light irradiance and ended at the highest.

Pharmacology

The glutamatergic blockers L (+)-2-amino-4-phosphonobutyrate (L-AP4) (group III metabotropic glutamate receptor agonist) (50 µM), 6, 7-dinitroquinoxaline-2, 3-dione (DNQX) (AMPA/kainate receptor antagonist) (40 µM), and d-2-amino-5-phosphonovalerate (d-AP5) (NMDA receptor antagonist; all from Sigma Aldrich, UK) (40 µM) were added to the AMES media, to block synaptic input from outer retinal photoreceptors and identify the origin

of light responses in retinal explants. Under these conditions, we repeated the 1s light pulses described above. Following this protocol, the drugs were washed out and the MEA chamber was cleared with AMES media (Sigma Aldrich, UK) for up to 1 hour before repeating the same 1 s light stimulation protocol.

Data analysis

Spike sorting

Offline, neural waveforms were processed using Offline Sorter (v4.7.1; Plexon). Cross-channel artifacts were identified and removed, and then each channel was analyzed separately. For each channel, single-unit spikes were detected and categorized based on the spike waveform via a principal component analysis, whereby distinct clusters of spikes were readily identifiable and showed a clear refractory period in their interspike interval distribution (>1 ms). Single-unit data were subsequently sent too and stored in NeuroExplorer (v5.437; Nex Technologies, MA) in preparation for further analysis.

Identification of light responses

Spike sorted data in Neuroexplorer files was analyzed by custom written MATLAB codes as reported previously (Ribeiro et al., 2021). The Peri-stimulus time histograms (PSTHs) was calculated with 100 ms bins (over 10 repeated trials). Thresholds were defined using both the amplitude and duration of responses. For increases in firing rate, this was defined as the pre-stimulus baseline +3 standard deviation (SD) with 100 ms duration, while for decreases in firing rate this was the pre-stimulus baseline 2 SD with 300 ms duration. Baseline is defined as the average firing rate in the 2s preceding the stimulus over 10 repeated trials. The rules used to assign each neuronal response to a specified class are described in full in (Ribeiro et al., 2021).

Latency & amplitude analysis

Amplitude and Latencies were calculated for individual light responsive units. Amplitude is defined as the change in firing rate between light onset/offset and peak response. Latency is the time difference between stimulus onset/offset and peak response. Latencies were calculated from smoothed PSTHs with 10ms bin to retain an appropriate time resolution, while amplitude was calculated with a 100ms bin. Latency was then plotted for different component of light-responsive units based upon their classification to the 1s light pulse as above and binned at 10ms. Latency to the ON component of responses (ON, ON Suppressed by Dark and ON-OFF units) was calculated as the time at which maximum firing rate was reached following light onset. Latency to the OFF component of responses (OFF, OFF Suppressed by Light and ON-OFF units) were calculated as the time at which maximum firing rate was reached following light offset.

Sensitivity analysis

Single units were filtered to ensure that the firing rate at the highest irradiance demonstrated a significant change in firing rate that was > 3 SD above the pre-stimulus baseline. If this criterion was met, the response of that unit at the eight lower intensities was used for analysis regardless of whether it crossed the confidence interval. Sensitivity curves were calculated by subtracting the pre-stimulus baseline from the average peak firing rate in response to the 100ms light step

Pharmacology Analysis

Single units were categorised to the 1s light step as defined above. The peak firing rate for each light responsive unit was then calculated under the Pharmacology and Washout

conditions. Peak firing rates were statistically analysed for each unit using a 2-Way ANOVA with Dunnett's Multiple Comparison Test.

Statistical analysis

All values are presented mean ± SD (standard deviation) unless otherwise stated; N, number of animals, retinas or independent experiments performed, where appropriate; n, number of cells or images examined, where appropriate. For MEA experiments, n = number of single units. Statistical significance was assessed using Graphpad Prism software and denoted as $p<0.05$ = *; $p<0.01$ = **; $p<0.001$ = ***. Appropriate statistical tests were applied including 2 tailed t-test (Mann Whitney), 1-way ANOVA with Tukey's correction for multiple comparisons, 2-way ANOVA with Bonferroni's correction, and paired and unpaired T-tests were used to compare latency and amplitude calculations. The test used in each experiment is reported in the Results section.

Figures were generated in Adobe Illustrator, Adobe Photoshop and CorelDraw.

SUPPLEMENTARY TABLES

Antibodies	Source
Rhodospin	Sigma
Mouse Cone Arrestin	Merck Millipore
S-Ospin	Merck Millipore
L/M Opsin	Merck Millipore
Peripherin-2 (PRPH2)	Merck Millipore
PKC-α	Santa Cruz Biotechnology
Calbindin	Swant
Secretagogin (SCGN)	Biovendor
Calretinin	Abcam
GFAP	Calbiochem
Human Cone Arrestin	Novus Biologicals

Human Nuclei	Merck Millipore
CtBP2 (Ribeye)	BD Biosciences
mGluR6	
Peanut agglutinin (PNA) biotinylated	2BScientific, B-1075-5
Donkey anti-mouse Alexa Fluor 488	INVITROGEN, ThermoFisher Scientific
Donkey anti-rabbit Alexa Fluor 405	INVITROGEN, ThermoFisher Scientific
Donkey anti-rabbit Alexa Fluor 546	INVITROGEN, ThermoFisher Scientific
Donkey anti-mouse Alexa Fluor 546	INVITROGEN, ThermoFisher Scientific
Donkey anti-chicken Alexa Fluor 647	INVITROGEN, ThermoFisher Scientific
Donkey anti-rabbit Alexa Fluor 647	INVITROGEN, ThermoFisher Scientific
Streptavidin Alexa Fluor 633 conjugate	INVITROGEN, ThermoFisher Scientific

Supplementary Table 1

Supplementary Table detailing primary and secondary antibodies used.
