# Supplementary material for: Human cone photoreceptor transplantation stimulates remodeling and restores function in AIPL1 model of end-stage Leber congenital amaurosis
Source: Stem Cell Reports. 2025 Mar 27;20(4):102470. doi: 10.1016/j.stemcr.2025.102470 (PMC12069896; doi:10.1016/j.stemcr.2025.102470)
Supplement: Document S2. Article plus supplemental information [file mmc3.pdf]

Stem Cell Reports
Article
OPEN ACCESS

Human cone photoreceptor transplantation stimulates remodeling and restores function in AIPL1 model of end-stage Leber congenital amaurosis

Christopher A. Procyk,[1] Anna Melati,[1] Joana Ribeiro,[1,2] Jingshu Liu,[1] Matthew J. Branch,[1] Jamie D. Delicata,[1] Menahil Tariq,[1] Aikaterini A. Kalarygrou,[1,3] Jessica Kapadia,[1] Majid Moshtagh Khorsani,[1] Emma L. West,[1] Alexander J. Smith,[1] Anai Gonzalez-Cordero,[1,4] Robin R. Ali,[1,5] and Rachael A. Pearson[1,5,6,*]

[1]Ocular Cell and Gene Therapy Group, Centre for Gene Therapy and Regenerative Medicine, King's College London, Guy's Hospital, London SE1 9RT, UK
[2]Present address: Evotec, Manfred Eigen Campus, Hamburg, 22419, Germany
[3]Present address: Harvard University, Massachusetts Eye and Ear Infirmary, Boston, MA 02114, USA
[4]Present address: Children's Medical Research Institute, University of Sydney, Westmead NSW 2145, Australia
[5]Senior authors
[6]Lead contact
*Correspondence: rachael.pearson@kcl.ac.uk
https://doi.org/10.1016/j.stemcr.2025.102470

SUMMARY

Photoreceptor degeneration is a leading cause of untreatable sight loss. Previously, we showed that human pluripotent stem cell-derived cone photoreceptors (hCones) can rescue retinal function in the *Rd1* mouse model of rod-cone dystrophy. However, retinal degenerations display markedly different severities and concomitant remodeling of the remaining retina; for photoreceptor replacement therapy to be broadly effective, it must work for a variety of disease phenotypes. Here, we sought to rescue the *Aipl1*$^{-/-}$ model of Leber congenital amaurosis, a particularly fast, severe condition. After transplantation of hCones, host cone bipolar cells underwent extensive remodeling and formed nascent synaptic-like connections. Electrophysiological recordings showed robust rescue of light-evoked activity across visually relevant photopic intensities, and treated mice exhibited visually evoked optokinetic head-tracking behavior. Thus, human cone photoreceptor replacement therapy is feasible even in very severe cases of retinal dystrophy, offering promise as a disease-agnostic therapy in Leber congenital amaurosis (LCA) and in other advanced retinal degenerations.

INTRODUCTION

In the later stages of retinal degeneration, patients suffer near-complete loss of photoreceptor cells and, consequently, blindness. To date, there are few treatment options for such advanced disease. We reported rescue of mouse models of rapid retinal degeneration using gene supplementation therapy (Nishiguchi et al., 2015). However, the speed of these degenerations offers only a short treatment window (before post-natal day [P]13 in mice). In patients with mutations in the *AIPL1* gene, the degeneration is typically nearly complete by 3 years of age, making early diagnosis and treatment essential but challenging (Tan et al., 2012). Consistent with this, a recent report indicated that, for effective gene therapy-mediated rescue of *CNGB1*-mediated photoreceptor degeneration, treatment must occur while >50% of photoreceptors remain (Scalabrino et al., 2023). Moreover, most gene therapy approaches are necessarily gene specific, and there is a need for disease-agnostic approaches (Klymenko et al., 2024). Alternative strategies for treating end-stage disease include electrical implants, which have demonstrated modest efficacy in clinical trials, and optogenetic approaches targeting the remaining retinal neurons, which have yielded promising pre-clinical results but presently lack photosensitivity within the normal physiological range (Parnami and Bhattacharyya, 2023), limiting their clinical utility.

Photoreceptor replacement therapy offers a physiological approach and has the potential for treating early-onset and/or severe diseases after photoreceptor loss. We have previously explored murine photoreceptor transplantation in several models of retinal degeneration (Barber et al., 2013; Gonzalez-Cordero et al., 2017; Pearson et al., 2012, 2016). More recently, we demonstrated that transplanted human stem cell-derived cones (herein referred to as "hCones") can form new contacts with retinal neurons in recipient *Rd1* mice that were backcrossed to *FoxN1nu* (*Rd1/FoxN1*^{nu}) to create an immunodeficient model suitable for receiving human cells and restore light-evoked retinal responses (Ribeiro et al., 2021). Such rescue was not necessarily expected. Rapid and extensive degeneration presents significant challenges: the inner retina undergoes substantial remodeling, including reduction or loss of both pre- and postsynaptic proteins at the outer plexiform layer and retraction and/or sprouting of interneuron axons and dendrites (Marc et al., 2003; Strettoi et al., 2003). Reactive gliosis is also common (Hippert et al., 2015; Matsuyama et al., 2022). Importantly, the extent of remodeling and concomitant glial scarring can differ markedly in different models of retinal degeneration impeding contact between donor and recipient retinal neurons (Barber et al., 2013; Hippert et al., 2015). Thus, it cannot be assumed that photoreceptor replacement can rescue all advanced diseases.

Published by Elsevier Inc. on behalf of International Society for Stem Cell Research.
This is an open access article under the CC BY license (http://creativecommons.org/licenses/by/4.0/).

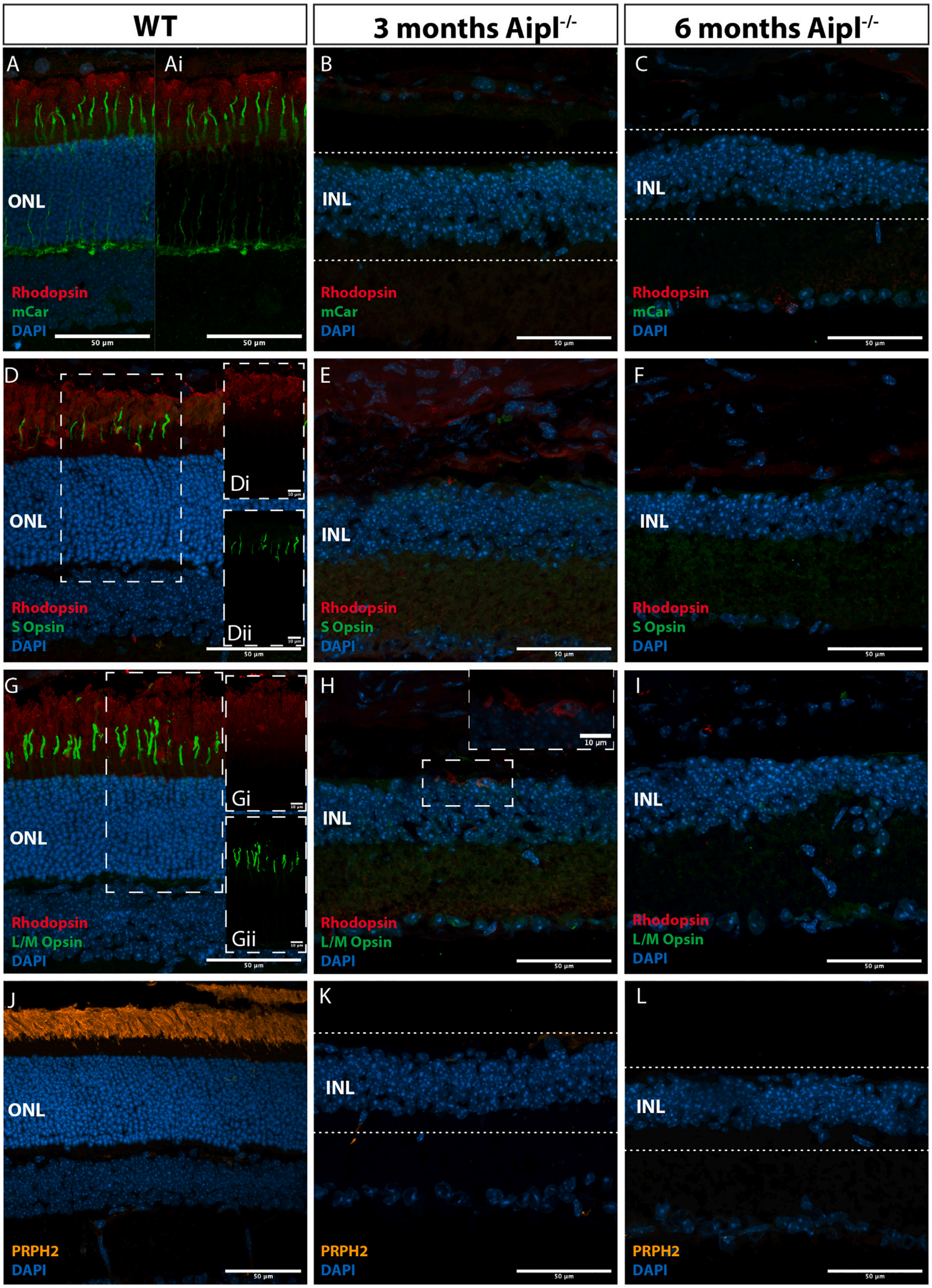

(legend on next page)

To support the wider applicability of photoreceptor replacement therapy, we sought to determine if it can affect meaningful functional rescue in an *Aipl1*$^{-/-}$/*FoxN1*^{nu} mouse model of LCA. The *Aipl1*$^{-/-}$ line is notable as it presents extremely rapid and widespread photoreceptor degeneration, beginning shortly after photoreceptors are born (Ramamurthy et al., 2004). Indeed, degeneration is so rapid that it affects the expression of postsynaptic markers, suggesting a failure or abnormal development of photoreceptor-bipolar cell (BC) synapses during synaptogenesis (Singh et al., 2014), presenting a particularly severe case for rescue.

RESULTS

Inner retinal remodeling after photoreceptor loss in the *Aipl1*$^{-/-}$ mouse retina

The broad pattern of degeneration in the *Aipl1*$^{-/-}$ model has been reported previously (Ramamurthy et al., 2004). Here, we backcrossed to *Fox1Nu* mice to create an immunodeficient model (herein called *Aipl1*$^{-/-}$) and characterized it in more detail, particularly regarding any remaining photoreceptors, pre- and postsynaptic proteins, and inner nuclear layer (INL) integrity at 3 (age at transplantation) and 6 months of age (age at assessment, 3 months post-transplantation) (Figures 1A–1L). Immunohistological examination of Rhodopsin confirmed that all rod photoreceptors had degenerated in the 3-month *Aipl1*$^{-/-}$, except for the very occasional cell body (Figure 1H), and mouse-specific Cone Arrestin (mCar) showed that all cone photoreceptors had degenerated within the central-mid retina by 3 months (Figures 1B and 1C), with very rare cell bodies observed in the periphery (Figures S1A and S1E). No labeling for S-opsin (Figures 1E and 1F); L/M-opsin (Figures 1H and 1I); or the structural outer segment protein, Peripherin-2 (Prph2; Figures 1K and 1L) was seen in the central-mid retina at either 3 or 6 months. The rare peripheral cones observed at 3 and 6 months lacked any discernible outer segment-like structures but retained some S- or L/M-opsin in the cell bodies (Figures S1B–S1D and S1F–S1H). Thus, the central retina of the *Aipl1*$^{-/-}$ is devoid of photoreceptors at the time of transplantation.

We next assessed the impact of this rapid outer nuclear later (ONL) loss on inner retinal neurons (Figure 2). Protein kinase C alpha (PKCα) labels both rod and cone ON BCs, while secretagogin (SCGN) labels cone (ON and OFF) BCs only (Puthussery et al., 2010). Both PKCα+ (Figures 2A–2C) and SCGN+ (Figures 2D–2F) dendrites exhibited substantial retraction in both 3- and 6-month-old *Aipl1*$^{-/-}$ mice. Notably, both PKCα+ and SCGN+ BCs maintained their axonal projections and synaptic pedicles, despite the absence of photoreceptor input. Calbindin+ horizontal cell (HC) dendrites (Figures 2G–2I) were also retracted, and, increasingly, their cell bodies appeared “flipped,” positioned apical, rather than basal, to the outer edge of the INL (Figure 2I, *arrow*). Amacrine (AC) and retinal ganglion cells (RGCs) were visualized by immunolabelling for Calretinin (Figures 2J–2L). In the WT, this label reveals evenly spaced ACs and three well-defined synaptic sub-lamina in the inner plexiform layer (IPL) (Figure 2J). Even at 3 months, several weeks after photoreceptors have died, the *Aipl1*$^{-/-}$ retina still contained remarkably well-defined Calretinin+ sub-lamina, but this starts to break down by 6 months (Figures 2K and 2L). Lastly, we assessed reactive gliosis, as shown by upregulation of glial fibrillary acidic protein (Gfap). As expected, Müller glia exhibited extensive Gfap expression, with ramified, hypertrophic apical processes extending along the outer limits of the INL by 3 months, persisting at 6 months (Figures 2M–2O).

The INL appeared thinner in *Aipl1*$^{-/-}$ mice, compared with the WT, and a previous report had indicated loss of PKCα+ BCs from 1 month onwards (Singh et al., 2014). We found that the number of PKCα+ BCs was slightly reduced in *Aipl1*$^{-/-}$, although this was not statistically significant, compared with age-matched WT controls (Figure 2P). SCGN+ BCs also decreased slightly by 6 months, but again this was not statistically significant (44.7 ± 2.8 vs. 38.6 ± 0.8, WT vs. *Aipl1*$^{-/-}$; two-way ANOVA; Figure 2Q). Calbindin+ HCs underwent significant loss by 6 months (2.9 ± 0.4 vs. 2.5 ± 0.4, WT vs. *Aipl1*$^{-/-}$ at 3 months and 3.2 ± 0.1 vs. 1.9 ± 0.3 at 6 months; $p < 0.05$, two-way ANOVA; Figure 2R).

We next assessed synaptic protein expression in the outer plexiform layer (OPL). Pre-synaptic Ribeye is essential for the formation of ribbon synapses and presents a horseshoe pattern of labeling in WT retina (Figure 2S). By 3 months, it is virtually undetectable in the *Aipl1*$^{-/-}$ retina (Figure 2T); very occasional puncta were detected, mis-localized to

Figure 1. Photoreceptor degeneration in the *Aipl1*$^{-/-}$ retina

Representative confocal maximum image projections (MIPs) for WT and 3- and 6-month *Aipl1*$^{-/-}$ (A–C) Mouse cone arrestin (*green*) and rhodopsin (*red*) staining of cone and rod photoreceptors, respectively.
(D–F) S-Opsin (*green*) and rhodopsin (*red*).
(G–I) M/L Opsin (*green*) and rhodopsin (*red*) immunostaining.
(J–L) Outer segment protein, Peripherin-2 (Prph2; orange).
Scale bar 50 μm, except ROIs of (D–H), 10 μm. ONL, outer nuclear layer; INL, inner nuclear layer; mCar, mouse cone arrestin; Prph2, Peripherin-2; WT, wild type. DAPI, nuclear label.

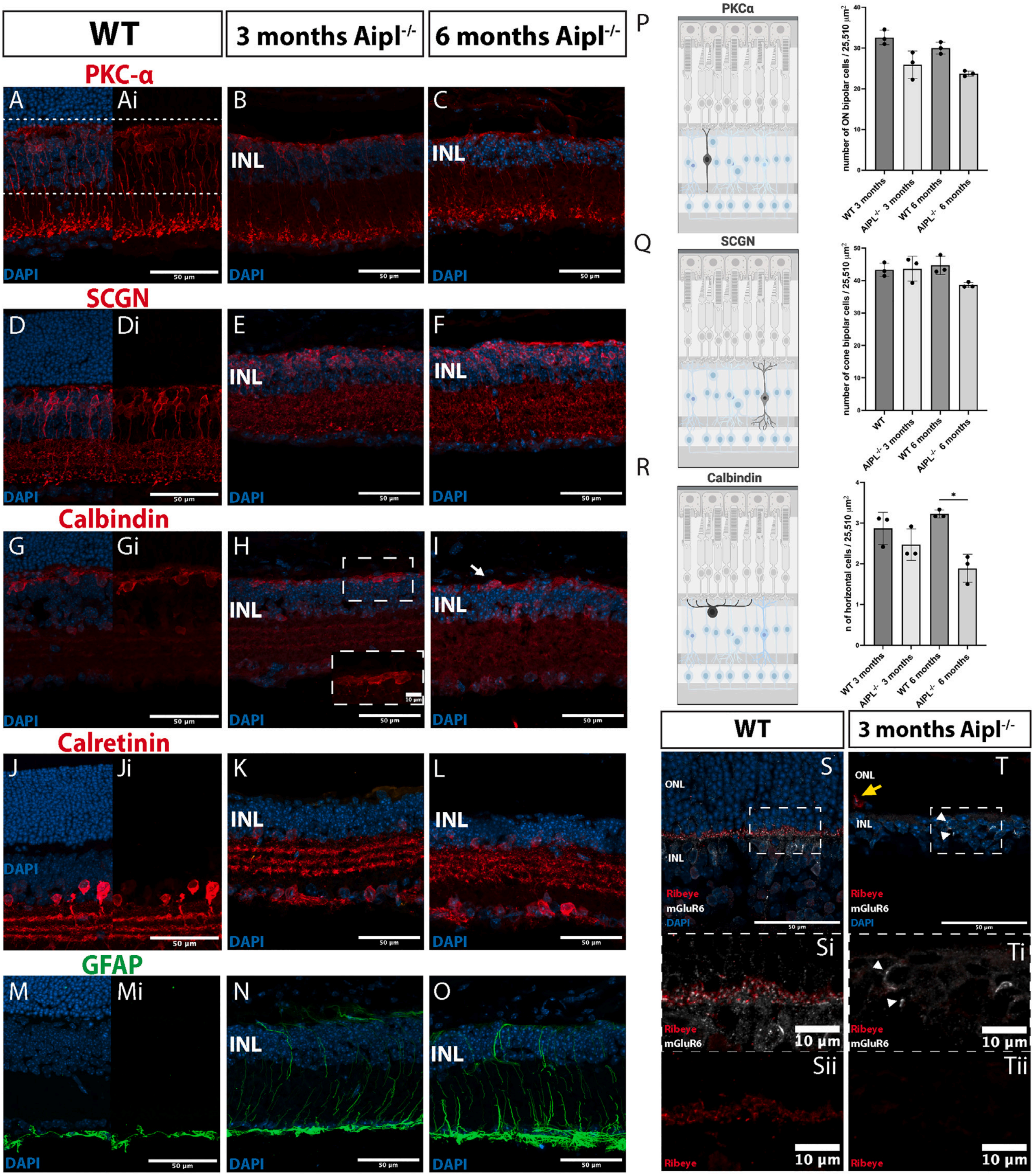

Figure 2. Inner retinal cell loss and dendritic remodeling in the *Aipl1*$^{-/-}$ retina

(A–O) Representative confocal MIPs of, from left to right in each row, 3-month-old WT, 3-month-old, and 6-month-old *Aipl1*$^{-/-}$ mice, immunostained for (A–C) PKCα-positive ON BCs, (D–F) Secretagogin (SCGN)-positive ON and OFF cone BCs, (G–I) Calbindin+ HCs, (J–L) Amacrine cells (ACs), stained for calretinin, and (M–O) reactive Müller glia and astrocytes. At both 3 and 6 months, both types of BCs and HCs show retraction of dendritic arborizations in the *Aipl1*$^{-/-}$. Some HCs "flip" the orientation of their soma toward the apical surface (white arrow, I). ACs show limited changes, with some disorganization of IPL sub-laminae by 6 months.

(legend continued on next page)

the cytoplasm (Figure 2T, *yellow arrow*). No Ribeye was seen at 6 months. The postsynaptic receptor, mGluR6, is expressed in BC dendrites and seen close to ribeye puncta in the WT (Figure 2S). mGluR6 is downregulated as photoreceptors die in degeneration (D'Orazi et al., 2014; Ribeiro et al., 2021), and expression was almost undetectable in *Aipl1*$^{-/-}$ by 3 months, seen only in the cell bodies of a few BCs, but this was not correlated with Ribeye expression (Figure 2T, *arrowheads).*

Transplanted human cones repopulate large areas of the *Aipl1*$^{-/-}$ host retina and mature to express phototransduction machinery

Previously, we transplanted 100,000 hCones/eye into the non-immunocompromized *Aipl1*$^{-/-}$ model and examined them 3 weeks post-transplantation; we observed fair donor cell survival but limited evidence of connectivity (Gonzalez-Cordero et al., 2017). In a more recent study in the *Rd1/FoxN1*^{nu} mouse, we increased donor cell number to 500,000/eye and examined rescue at 3 months post-transplantation, finding good survival and maturation of donor cells and rescue of light-evoked retinal and behavioral responses (Ribeiro et al., 2021). We therefore followed the same approach here, transplanting 500,000 hCones/eye in 3-month-old immunocompromized *Aipl1*$^{-/-}$ mice and examining 3 months post-transplantation. Human cones were pre-labeled in the organoid using adeno-associated virus (AAV)-ShH10(Y445F) containing GFP under the control of the 2.1 L/M-opsin promoter (L/M-opsin.GFP) (Gonzalez-Cordero et al., 2017, 2018; Ribeiro et al., 2021) and sorted before transplantation.

L/M-opsin.GFP+ hCones exhibited good survival and formed a layer of variable thickness, but typically 10–15 cell bodies thick, immediately adjacent to the host INL (Figures 3A–3H). The area covered varied between transplants but averaged 2.48 mm^2 (±0.36; n = 4 eyes), based on flat-mount preparations. We confirmed these cells were of human origin by staining for human nuclei antigen (HNA) (Figure 3A) and measuring nuclei size, which were significantly larger than mouse cone nuclei, as determined by measuring GFP+ cones within adult *Chrnb4.eGFP* mice (Figure 3C). The HNA+ cells also widely expressed human-specific cone arrestin (hCAR) (Figure 3A), and many PRPH2+ buds were observed within the cell mass (Figure 3B).

The host inner retina undergoes substantial remodeling, and cone BCs make synapse-like contacts with transplanted human cones

We examined how *Aipl1*$^{-/-}$ inner retinal neurons responded to the presence of healthy hCones. PKCα+ (ON) BCs re-elaborated extensive dendritic arborizations, sending them throughout the donor cell mass (Figure 3E), a feature seen exclusively in areas where hCones were present (Figures S2A–S2Aii). Notably, some PKCα+ cell bodies were also seen within the donor cell mass, apparently migrated from the INL (*white arrowheads*, Figures 3D–3Dii). Despite many studies being interested in cone-mediated rescue, few (including our own) have examined interactions between donor cones and host cone BCs specifically. SCGN+ cone BCs also re-extended dendrites into the donor cell mass (Figures 3D, 3F, and S2B); qualitatively, this appeared less extensive than PKCα+ dendrites (compare with Figure 3Ei), although this may reflect staining differences. Of note, SCGN+ cone BC cell bodies rarely migrated into the donor cell mass, unlike the PKCα+ population, suggesting this might be a rod BC-specific response.

The response of HCs to transplantation has received only very limited exploration to date (Akiba et al., 2024). Calbindin+ HCs also showed dendritic elaboration (Figures 3G and S2C), while calretinin+ ACs and RGCs appeared broadly unchanged (Figure 3H), compared with controls. Finally, since cones can recycle their visual pigments via Müller glia, we examined how host glial cells interacted with hCones. Co-staining with HNA showed that Gfap+ Müller glial processes originated from the host retina and were not contaminants from donor cell sorting; these processes were clearly visible throughout the host retina and extended into the donor cell mass. Indeed, in many instances, they appeared to delineate the apical-most boundary, this time above the transplanted hCones (Figure S3), thus incorporating them within the host neural retinal structure.

Next, we examined the expression of synaptic markers. Punctate labeling for the ribbon synapse protein, RIBEYE, was seen through the cell mass (Figure 4), including within processes extending toward the host inner retina. Both SCGN+ cone BCs and PKCα+ ON-type rod and cone BCs showed widespread re-expression of the postsynaptic marker, mGluR6 (Figure 4). Careful examination of RIBEYE

(P–R) quantification of PKCα+, SCGN+, and Calbindin+ cells in WT and in 3- and 6-month-old *Aipl1*$^{-/-}$. *$p < 0.05$, 2-way ANOVA, mean ± SD.
(S–Sii) Immunostaining for pre-synaptic Ribeye (*red*) and postsynaptic mGluR6 (*white*). Ribeye exhibited a typical "horseshoe" pattern and co-localizing with mGluR6 (MIP of 3 z sections).
(T–Tii) By 3 months of age in *Aipl1*$^{-/-}$ retinas, mGluR6 is translocated back to the cell bodies of BCs (*white arrows*), and no Ribeye labeling was detected in central retina (MIP of 3 z sections).
Scale bars: (A–O), 50 μm; (S and T), 50 μm; and (Si, Sii, Ti, and Tii), 10 μm. WT, wild type; INL, inner nuclear layer; SCGN, secretagogin; Gfap, glial fibrillary acidic protein.

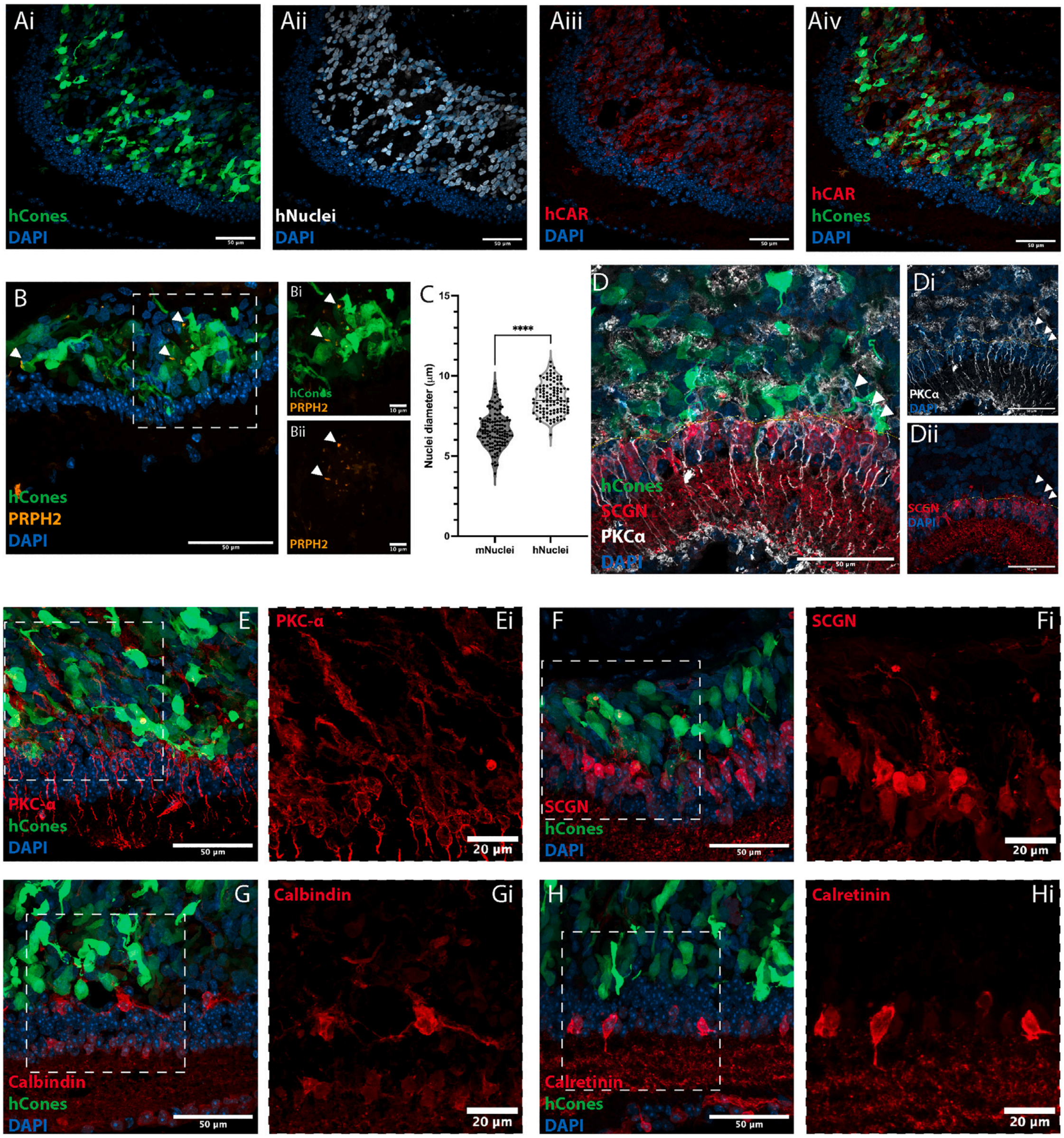

Figure 3. Human cones survive, mature, and promote re-extension of inner retinal neuron dendrites in the *Aipl1*$^{-/-}$ host retina

Representative confocal MIPs of *Aipl1*$^{-/-}$ retina 3 months post-transplantation.

(A) Large numbers of GFP+ hCones (*green*) could be seen in the subretinal space of 6-month *Aipl1*$^{-/-}$ mice. These cells labeled for (Aii) human nuclear antigen (HNA; labeled hNuclei; *gray*) and (Aiii) human cone arrestin (hCAR; *red*). (Aiv) All cells in cell mass were positive for HNA and hCAR staining, and all GFP+ cells labeled for both markers.

(B) GFP+ hCones co-localized with PRPH2+ bud-like structures, indicative of nascent segments (Bi and Bii white arrows).

(C) Quantification of the maximum diameter of murine cone (m)Nuclei, assessed in *Chrnb4.Gfp* mice, compared to hNuclei showed a significant difference in size (6.6 ± 1.1 μm vs. 8.6 ± 0.95 μm, $N = 3$ retina; $n = 127$ and $N = 3$, $n = 110$ nuclei respectively, $****p < 0.001$, Mann-Whitney test, mean ± SD).

(D–Di) Some PKCα+ ON BCs migrated into the cell mass (*white arrows*), while (Dii) SCGN+ cone BCs remain positioned in the INL.

(E–H) INL remodeling after hCone transplantation. Both PKCα+ and SCGN+ BCs and Calbindin+ HCs show extensive neurite elongation toward the transplanted cell mass.

Scale bars: (A–H), 50 μm; (Bi, Bii, Ei, F, Gi, and Hi), 10 μm. PRPH2, Peripherin-2; SGCN, Secretagogin, DAPI, nuclear label.

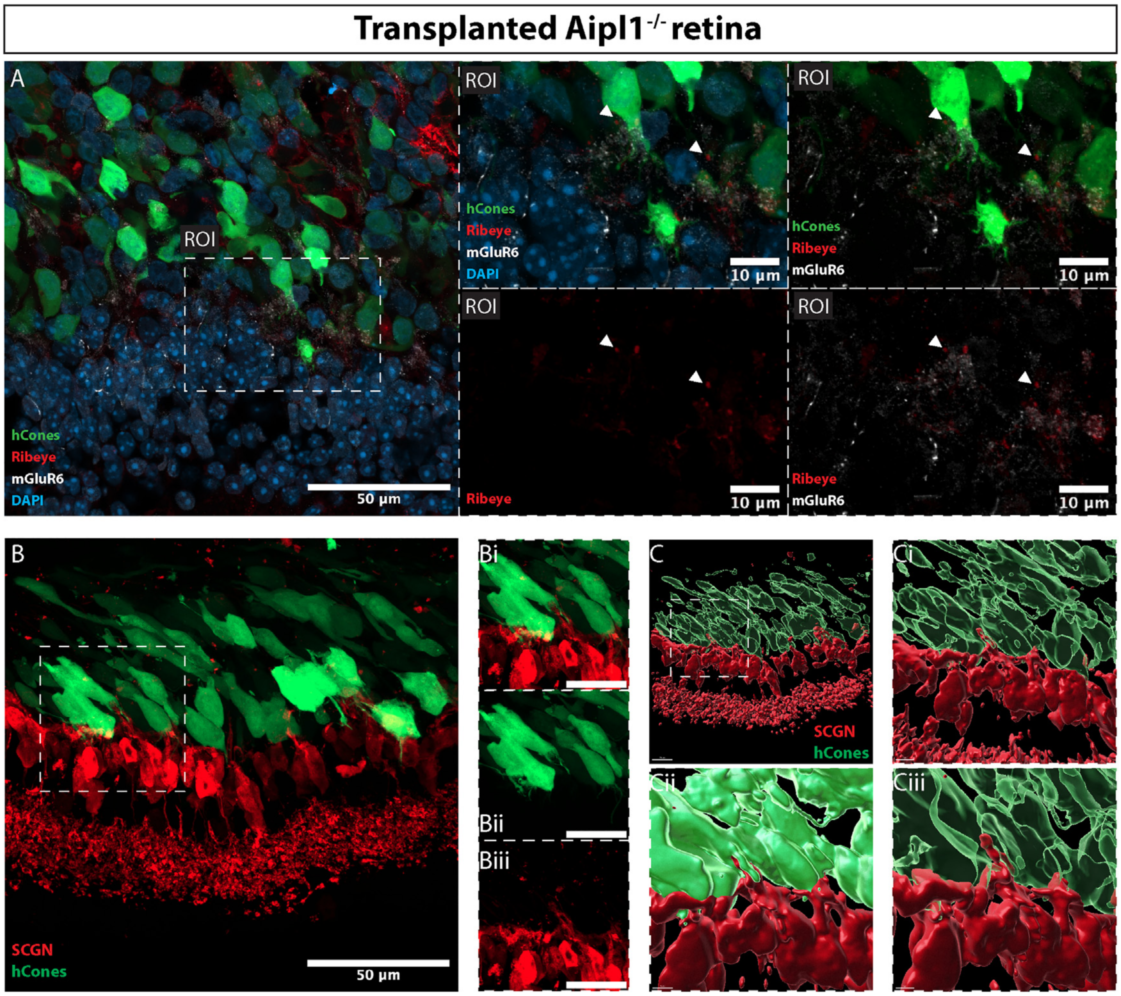

Figure 4. Human cones promote re-expression of postsynaptic mGluR6 and form nascent synaptic-like contact points with host BCs

Representative confocal MIPs of *Aipl1*$^{-/-}$ retina 3 months post-transplantation.

(A) Punctate labeling for mGluR6 (*gray*) was present throughout the area of transplant (ROI). RIBEYE+ (*red*) structures, present in transplanted GFP+ hCones (*green*), could be found near mGluR6+ structures (ROI).

(B) SCGN dendrites extend toward the cell mass, invaginating into the transplanted cones (Bi and Bii).

(C) IMARIS 3D reconstruction of cell surfaces of SCGN+ cells and transplanted cones highlight the contact made by SCGN+ dendrites to cone cell bodies (Ci, Cii, and Ciii).

Scale bar: (A and B), 50 µm; ROI, 10 µm; (Bi, Bii, and Biii), 20 µm. SCGN, Secretagogin, DAPI, Nuclear label

and mGluR6 revealed putative synaptic contacts (Figure 4, regions of interest [ROIs]); interestingly, 3D reconstruction of host dendrites and donor hCones indicated axo-somatic and dendro-somatic, as well as more typical axo-dendritic, contact points (Figures 4B and 4C and Video S1). Together these histological analyses show that hCones mature within the host *Aipl1*$^{-/-}$ environment, develop nascent, if imperfectly formed, structures required for phototransduction and synaptic transmission, and show morphological evidence of integration with the interneurons of the recipient retina.

Transplanted human cones rescue optokinetic head tracking but not ERG function in the *Aipl1*$^{-/-}$ model of advanced degeneration

Electroretinography (ERG) provides a gross measure of light-mediated trans-retinal function, averaged across the whole retina. Three *Aipl1*$^{-/-}$ mice receiving transplants underwent full-field photopic light flash (from dark) ERG recordings at 3 months post-transplantation. No repeatable, measurable response was seen, like uninjected *Aipl1*$^{-/-}$ control eyes (N = 3 animals). Positive controls were *Gnat1*$^{-/-}$ mice (N = 3 animals), which lack rod α-transducin, rendering rods

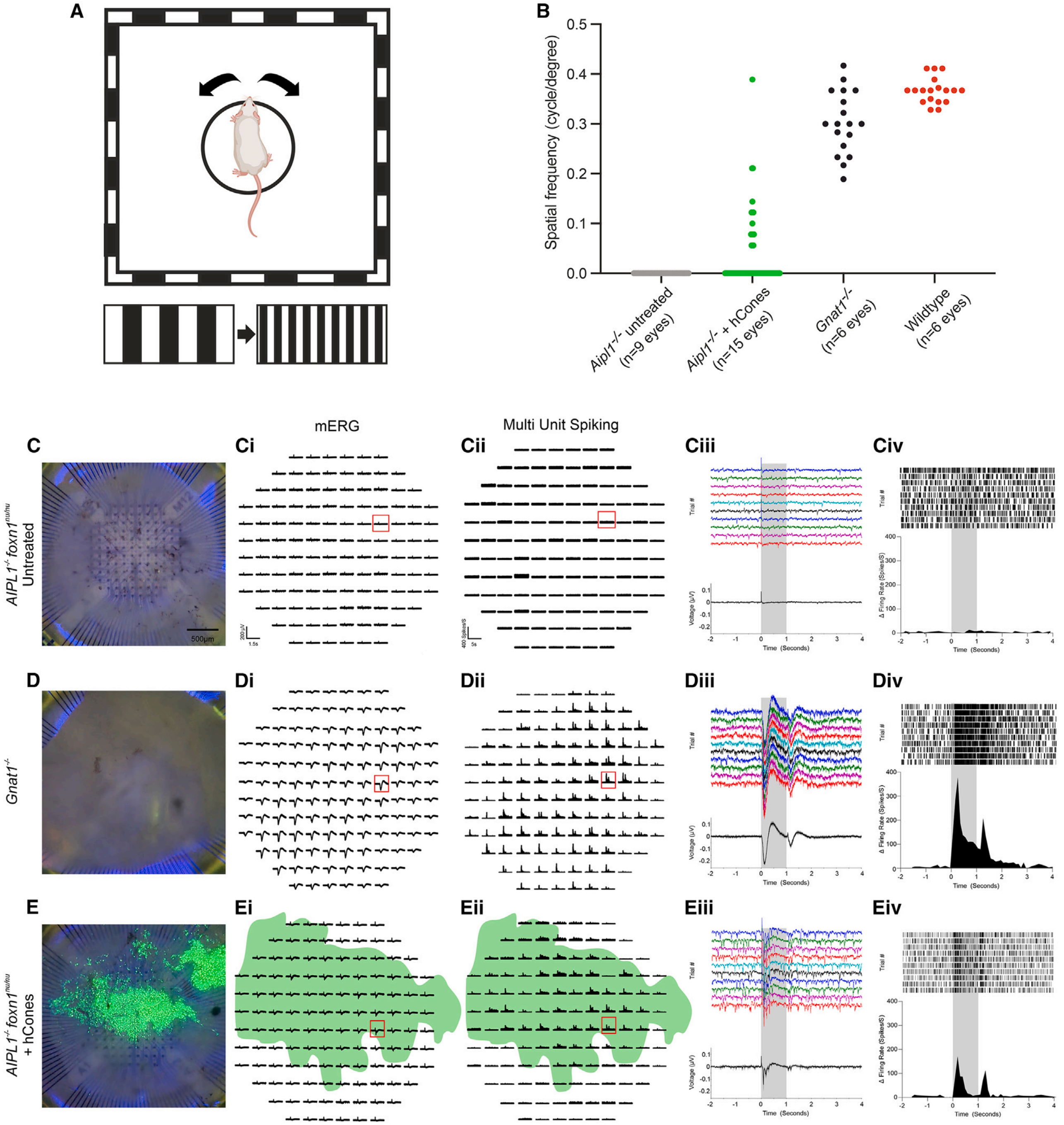

Figure 5. Transplantation of human cones restores optokinetic head-tracking behaviors and generates widespread mERGs and light-evoked spiking activity in the *Aipl1*$^{-/-}$ retina

(A) Schematic of OptoDrum optokinetic head-tracking setup.

(B) Scatterplot of all acuity measurements for untreated and hCone-transplanted *Aipl1*$^{-/-}$ eyes. *Gnat1*$^{-/-}$ and *C57Bl/6* WT mice are shown for comparison.

(C) Representative untreated *Aipl1*$^{-/-}$ retina on the MEA. (Ci) There were no discernible light-evoked mERGs nor (Cii) light-responsive spiking activity across the retina. Red boxes in (Ci and ii) indicate the representative trace magnified in (Ciii) mERG for 10 individual trials of a 1 s light pulse with the mean (±SD) below, and (Civ) the raster plot for MU spiking activity (*top*) and PSTHs (*below*) show no response to stimulus presentation. N.B. Photoelectric effect present at t = 0 in mERGs is due to the thinness of the *Aipl1*$^{-/-}$ retina.

(legend continued on next page)

non-functional but viable, and all photoreceptor-driven light responses are cone derived (Calvert et al., 2000). As expected, these presented robust responses to light flashes in the photopic range (Figure S4). This indicates that, if functional, a patch of transplanted hCones covering an area of ~6mm^2 (average detachment diameter is 2.5–2.8 mm), is insufficient to drive a reproducible full-field ERG response in mice.

To address whether transplantation can bring about changes in light-evoked behavior, we next measured optomotor head-tracking responses to a rotating grating (Figure 5A) (Pearson et al., 2012). The OptoDrum is a fully automated system, which permits independent visual threshold measurements from both eyes. Mice were assessed at 6 months of age (3 months post-transplant, where relevant). Each mouse was tested on 3–4 separate occasions within one week. Individual mice received injections to both eyes, or an injection to one eye only, or no injection. No head-tracking behavior was mediated by any uninjected *Aipl1*$^{-/-}$ eyes (N = 9), creating a very clean baseline. However, after transplantation, visual thresholds were measurable in 8/15 hCone-injected eyes, although these were not detectable in every recording session (e.g., visual threshold measurements may be determined in some but not all trials for a given mouse). Therefore, Figure 5B shows the distribution of all recorded thresholds across all treated and untreated eyes; there was a significant difference in the acuity thresholds measured in eyes receiving hCones, compared with uninjected *Aipl1*$^{-/-}$ eyes (p = 0.016, unpaired t test). Responses from WT and cone-only *Gnat1*$^{-/-}$ mice are shown for comparison.

Transplantation of human cones restores light-evoked mERGs and spiking activity in the host *Aipl1*$^{-/-}$ retina

After optomotor and ERG recordings, mice were euthanized and the retinas were carefully dissected prior to multi-electrode array (MEA) recording. Light responses were evoked using a 1 s uniform light step from darkness, and microERG (mERG) and multi-unit (MU) spiking activity were recorded from the ganglion cell layer (GCL) (Figures 5C–5E). Untreated *Aipl1*$^{-/-}$ retinas exhibited no discernible mERG in response to the light step and no change in firing rate following light onset or offset when observing the peristimulus time histogram (PSTH) (Figures 5C–5Civ; N = 3 retinas). In comparison, *Gnat1*$^{-/-}$ retinas (Figures 5D–5Div; N = 4 retinas) showed a large-amplitude mERG with both a discernible a- and b-wave, in response to light. MU firing also demonstrated a diverse array of fast transient light responses at both light onset and/or offset. The same light stimulus was presented to hCone-transplanted *Aipl1*$^{-/-}$ (Figures 5E–5Eiv; N = 4 retinas) and in all cases yielded robust and reproducible mERGs from the electrodes immediately under the GFP+ hCone donor cell mass. This correlated strongly with fast transient increases in MU firing, which were time locked to stimulus onset and/or offset and restricted to regions under the cell mass.

Sensory characteristics of visual responses

We determine the sensory characteristics of RGC responses using spike sorting. Human cone-transplanted *Aipl1*$^{-/-}$ retinas showed a significantly higher proportion of light-responsive units (53.4% ± 9.9%), compared with untreated *Aipl1*$^{-/-}$ retinas, (3.7% ± 0.7%; p = 0.0015); not unexpectedly, this was lower than that found in fully intact *Gnat1*$^{-/-}$ retinas (88.5% ± 2.0%; p = 0.0074) (Figure 6A). We next classified the single units based on their PSTH in response to a 1 s light step, as previously described (Ribeiro et al., 2021). The mean (±SEM) PSTH of these 10 classifications is shown for *Gnat1*$^{-/-}$, *Aipl1*$^{-/-}$ + hCones and untreated *Aipl1*$^{-/-}$ (Figure 6B). Comparing the cohorts (Figures 6C and 6D), the greatest proportion of responses observed were ON responses (increased firing rate at light onset) (65% in *Aipl1*$^{-/-}$ + hCones, compared with 23% in *Gnat1*$^{-/-}$), and fewer were ON-OFF responses in *Aipl1*$^{-/-}$ + hCones transplanted retinas, compared with *Gnat1*$^{-/-}$ controls (21% vs. 61%, respectively), although the overall proportion of OFF-only responses remained similar between the two genotypes (10% and 11%, respectively). Of the few channels responding to light in untreated *Aipl1*$^{-/-}$ mice, all classified as slow sustained responses, typical of the deafferented retina (Procyk et al., 2015).

(D) Representative *Gnat1*$^{-/-}$ retina on the MEA. (Di) Most channels exhibit large-amplitude light-evoked mERGs with a clearly defined a- and b-wave. (Dii) MU spiking activity was observed across the spatial extent of the array with a wide variety of increases and/or decreases at light onset and/or offset. (Diii) Magnified trace from the red box in (Di) and (Dii) shows reproducible mERG across individual trials and mean (±SD) (*bottom*), which are time locked to stimulus onset and offset. (Div) The same channel shows transient, large-amplitude changes in firing rate at light onset and/or offset in both the raster plot and PSTH across 10 individual trials.

(E) Representative transplanted retina. (Ei) mERGs are seen on large proportion of channels and correlate with position of GFP$^+$ cell mass (*green overlay*). (Eii) PSTH of MU spiking activity demonstrated a wide variety of increases and/or decreases at light onset and/or offset on channels correlating with the position of the GFP$^+$ cell mass (*green overlay*). Magnified trace of red box within the cell mass in (Ei) and (Eii) shows (Eiii) discernible and reproducible mERG time locked to stimulus onset/offset and (Eiv) the raster plot and PSTH for a channel that shows increases in firing rate at both light on/offset.

Scale bars: (C–E), 500 μm; (Ci, Di, and Ei), 200 μV and 2 s; (Cii, Dii, and Eii), 400 spikes/s and 4 s. Gray bars denote 1 s light pulse in (Ciii), (Civ), (Diii), (Div), (Eiii), and (Eiv).

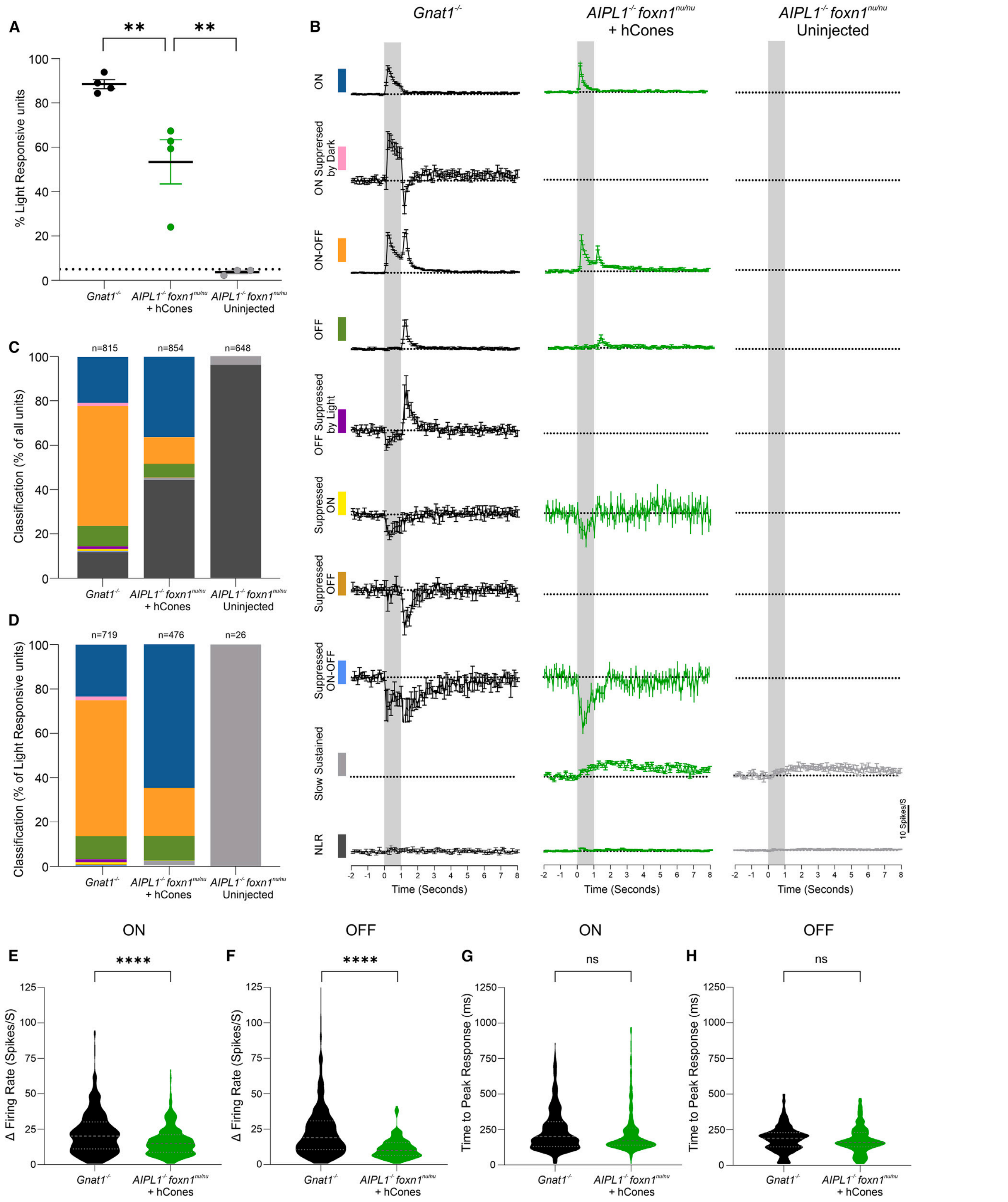

(legend on next page)

We next examined peak response amplitude and latency. Unsurprisingly, given the histological indications of limited outer segment extension, the average response amplitude was significantly lower for both the ON and OFF responses in *Aipl1*$^{-/-}$ + hCone-transplanted retinas, compared with *Gnat1*$^{-/-}$ cone-only controls ($p < 0.001$ for both; unpaired t test) (Figures 6E and 6F). Importantly, however, the peak response latency in *Aipl1*$^{-/-}$ + hCone-transplanted retinas for both ON and OFF responses was similar to *Gnat1*$^{-/-}$ (Figures 6G and 6H; $p = 0.306$ and $p = 0.29$ for ON and OFF, respectively; unpaired t test), indicating that the transmission of visual information from the transplanted human cones to the host RGCs is within the normal range.

Light-evoked RGC responses are mediated via glutamatergic transmission from transplanted human cones

To confirm that the light-evoked responses recorded in hCone-transplanted retinas originate from the hCones via glutamatergic transmission, we repeated the 1 s light stimulus protocol before, during, and after exposure to synaptic blockers (L-2-amino-4-phophonobutyric acid, L-AP4; 6,7-dinitroquinoxaline-2,3-dione, DNQX; and D-2-amino-5-phosphonopentanoate, D-AP5). These drugs block transmission of all known visual information at the photoreceptor/BC synapse (Wong et al., 2007; Zhao et al., 2014). Application of synaptic blockers reversibly eradicated both the mERG and all fast, transient responses at light onset and/or offset in both *Gnat1*$^{-/-}$ retinas and hCone-transplanted *Aipl1*$^{-/-}$ retinas. Representative mERGs and PSTHs are shown for each genotype under each condition in Figure S5, while the mean (±SEM) PSTH for all ON and ON-OFF single unit responses before, during, and after drug application is shown in Figure S6, together with peak response amplitudes.

Transplanted human cones demonstrate increased response amplitude and latency at behaviorally relevant light levels

Finally, we sought to characterize the sensitivity of the observed visual responses, compared to normal photopic vision. We presented transplanted retinas with 100 ms flash stimuli across 7 log units of illumination. We identified 723 units that showed a significant increase in firing rate following light onset at the highest irradiance in the *Gnat1*$^{-/-}$ population and 380 units in the *Aipl1*$^{-/-}$ + hCone population. The mean (±SEM) PSTH of all light-responsive units at each irradiance is shown in Figure 7A along with the respective channels mERG (Figure 7A, *inset*). Figures 7B and 7C show the mean (±SEM) mERG a- and b-wave amplitudes at increasing irradiance, while the maximum change in firing rate and time to peak response are shown in Figures 7D and 7E, respectively. The transplanted *Aipl1*$^{-/-}$ retinas showed significant changes in peak firing rate over a wide range of light levels, like *Gnat1*$^{-/-}$ retinas, demonstrating that, within the transplanted area, hCones are operating across a substantial range of physiologically relevant irradiances. The transplanted retinas closely tracked the irradiance/response relationship of *Gnat1*$^{-/-}$ preparations up to 10^{14} photons/cm^2/s, showing a reduced change in firing rate thereafter. Combined, the increase in a-wave amplitude demonstrates that the donor hCones can amplify and encode light information with increasing irradiance, while the increases in b-wave amplitude and RGC spiking show that the host BCs and RGCs retain the ability to encode and transmit this visual information through the remnant neural retina following remodeling.

DISCUSSION

Photoreceptor replacement therapy is proposed as a potentially disease-agnostic treatment for reversing sight loss in advanced retinal disease. To fulfill this potential, photoreceptor replacement therapy must be shown to be effective in multiple models of advanced retinal degeneration. Here, expanding on our previous report examining the *Rd1* model (Ribeiro et al., 2021), we chose a particularly severe form of LCA, the *Aipl1*$^{-/-}$ mouse, which shows photoreceptor loss even before the first synapse with second-order

Figure 6. Transplanted human cones drive an array of fast, large-amplitude visual responses in the *Aipl1*$^{-/-}$ retina

(A) Percentage of light-responsive units in *Aipl1*$^{-/-}$ + hCone-transplanted retinas ($N = 4$) was lower than *Gnat1*$^{-/-}$ ($N = 4$) but significantly higher than untreated *Aipl1*$^{-/-}$ retinas ($p < 0.001$; one-way ANOVA).

(B) Average PSTH of single units categorized into 10 quantitatively defined types based on their response to a 1 s light step in *Gnat1*$^{-/-}$, *Aipl1*$^{-/-}$ + hCone and untreated *Aipl1*$^{-/-}$. Time bin, 0.1 s; gray bars illustrate duration of light pulse.

(C and D) (C) Distribution of light-response types as percentage of all single units and (D) as percentage of all light-responsive units. Response types are color coded, as in (B).

(E and F) Violin plots of response amplitude in *Aipl1*$^{-/-}$ + hCone retinas (E) for ON-type responses (16.70 ± 0.5 spikes/s; $n = 410$) and (F) OFF-type responses (11.65 ± 0.7 spikes/s; $n = 156$) were significantly smaller than in *Gnat1*$^{-/-}$ retinas (22.42 ± 0.6 spikes/s, $n = 621$ and 23.61 ± 0.8 spikes/s, $n = 525$; $p < 0.0001$ for both; unpaired t test).

(G and H) Violin plots of latency to peak response for ON and OFF components of light responses. Latency for ON-type responses in *Aipl1*$^{-/-}$ + hCone retinas (236.0 ± 8.3 ms) was not significantly different compared with *Gnat1*$^{-/-}$ retinas (246.4 ± 6.1 ms; $p = 0.31$, unpaired t test) or for OFF-type responses (179.1 ± 7.8 ms versus 188.1 ± 4.1 ms; $p = 0.29$, unpaired t test).

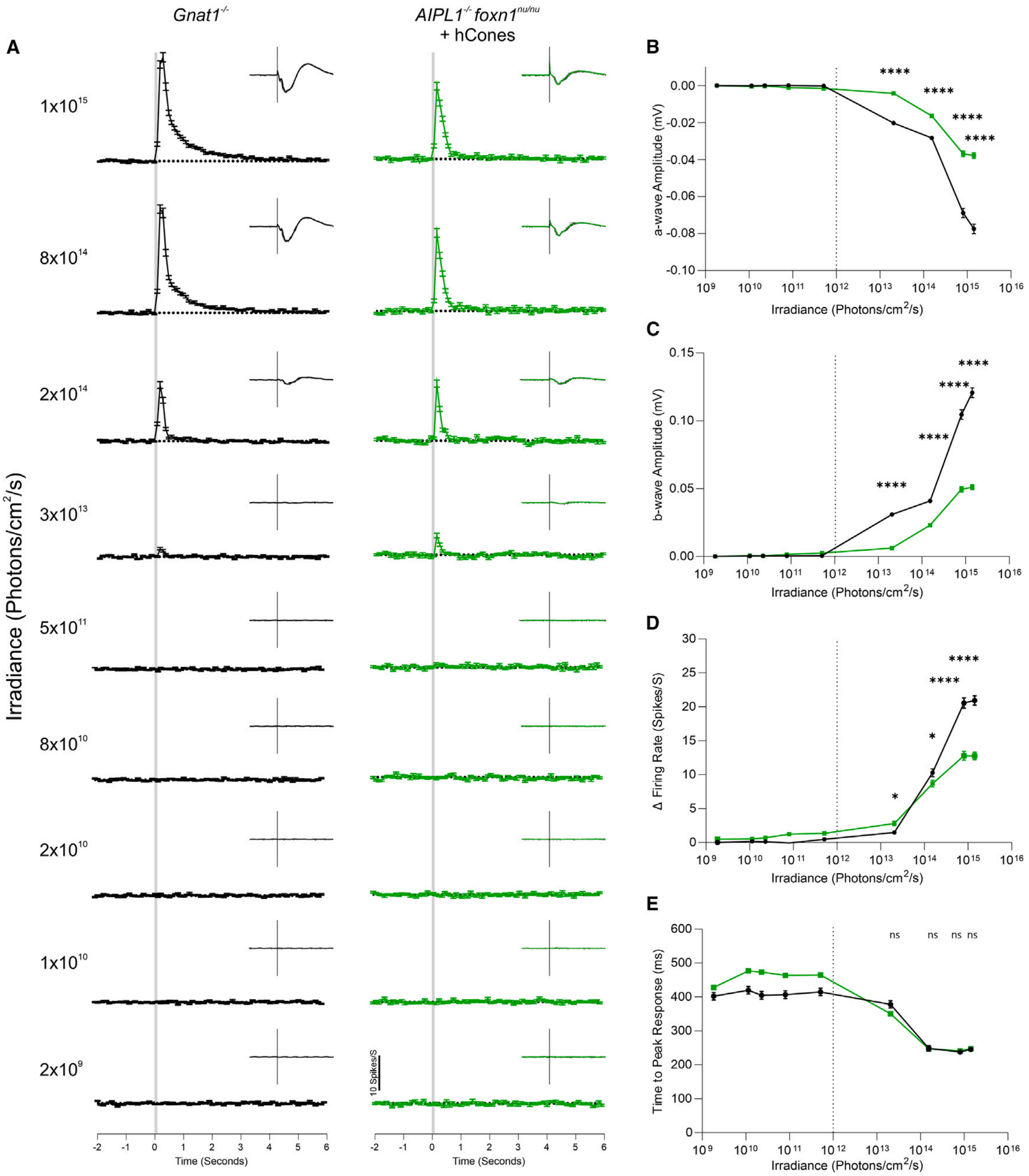

Figure 7. Increasing irradiance increases response amplitude in *Aipl1*$^{-/-}$ + human cones transplanted retinas

(A) Mean ± SEM PSTH of light responses in *Gnat1*$^{-/-}$ (*black*; *N* = 4) and *Aipl1*$^{-/-}$ + hCone-transplanted (*green*; *N* = 3) retinas across 9 increasing irradiances with (inset) corresponding mean ± SEM mERG of light-responsive channels (*n* = 480 and *n* = 281 channels, respectively).

(B and C) Mean ± SEM of mERG (B) a-wave and (C) b-wave amplitudes increase with increasing irradiance for both, from approximately 10^{12} photons/cm^2/s (vertical gray dotted line). *Gnat1*$^{-/-}$ showed a larger amplitude at each of the measurable irradiances above this level ($p < 0.001$).

(D) Mean ± SEM peak response amplitude of light-responsive units increases in both *Gnat1*$^{-/-}$ (*n* = 723 units) and *Aipl1*$^{-/-}$ + hCone-transplanted retinas (*n* = 380 units) above approximately 10^{12} photons/cm^2/s. Above this range, *Gnat1*$^{-/-}$ retinas demonstrates a

(legend continued on next page)

neurons is formed (Ramamurthy et al., 2004). This allowed us to directly address a key concern around whether a substantially remodeled inner retina can accept new inputs from healthy photoreceptors (Marc et al., 2003; Strettoi et al., 2003).

At the anatomical level, the untreated *Aipl1*$^{-/-}$ retina exhibited rapid and widespread photoreceptor loss; substantial downregulation of postsynaptic mGluR6 and dendritic retraction by rod and cone BCs in the INL; and a slow, progressive loss of those same cells over time. Other studies have indicated the potential for BCs to remodel after acute photoreceptor loss. In rabbit retina, laser ablation of photoreceptors leads rod BCs, but not cone BCs, to remodel to form new synapses with healthy photoreceptors outside the lesion (Beier et al., 2017), while, in adult primate retina, the dendrites of ON and OFF midget BCs at the border of a laser-induced scotoma also rewired to surviving photoreceptors (Akiba et al., 2024). Here, in the *Aipl1*$^{-/-}$ mouse retina, we find that, even several months after photoreceptor loss, both rod and cone BCs responded to the introduction of healthy human cones, with both PKCα+ and SCGN+ BCs re-extending previously retracted dendrites into the donor cell mass to form new contacts and re-express mGluR6. This indicates retention of a significant degree of anatomical plasticity by the remaining inner retinal neurons.

Retinal electrophysiology, as assessed using MEA recordings, yielded a wide variety of visual response types (ON, OFF, and ON-OFF), indicating that parallel processing pathways are still functional, consistent with the histological analysis. It is notable that a greater proportion of the responses observed were ON responses, and fewer were ON-OFF responses in transplanted retinas, compared with *Gnat1*$^{-/-}$ cone-only controls, while the overall proportion of OFF-only responses remained similar between the two genotypes. This may reflect the anatomical observations made recently by Wong and colleagues (Akiba et al., 2024), indicating that the midget ON pathway was maintained for longer than the OFF pathway in the region of laser ablation in the primate retina. One caveat is that the stimulus used in these experiments involves a light step from darkness, which may bias toward us finding ON-type responses. In future, challenging the transplanted retina with more naturalistic visual stimuli, such as steps of increasing and decreasing contrast from light-adapted backgrounds, may help identify a wider range of temporal response profiles, including OFF and suppressed responses, which would support the idea that the transplanted retina can encode a variety of both increases and decreases of irradiances at different light levels.

Whether the connections formed between transplanted donor photoreceptors and host inner retinal neurons represent a classic ribbon synapse has yet to be fully determined. The inevitable disorganization of where connections are made in such transplants makes it difficult to rely on anatomical location for validation. It is possible that the contacts made are nascent versions of a synapse or indeed atypical contacts; transsynaptic tracing methods could prove very interesting in this regard and for mapping the relative contributions of ON and OFF circuits in the restored connectome. Nonetheless, the temporal properties of the responses, together with the pharmacology, indicate a glutamatergic vesicular release-based mechanism in close proximity to postsynaptic mGluR6 receptors. Importantly, responses were also observed across a wide range of irradiances that fell within normal daylight levels, and the response times were again within the normal range for all but the very brightest of stimuli.

In our previous study transplanting hCones into the *Rd1* model of advanced retinal degeneration, we were unable to assess optokinetic head-tracking behaviors as the OptoDrum software encountered difficulties correctly detecting the position of the nude mouse. Adaptations to the software meant we were able to achieve this with the *Aipl1*$^{-/-}$/mouse model used here and show for the first time human cone-mediated improvements in optokinetic head tracking. Note that the maximum irradiance at the platform where the mouse is placed within the OptoDrum setup is limited to 8.8×10^{13} photons cm^{-2} s^{-1}. Examination of the irradiance response curves generated from the MEA recordings (Figure 7B) shows this to be at the onset of increasing RGC firing rates, and *Gnat1*$^{-/-}$ cone-only mice presented with reduced sensitivity on the optomotor, compared to WT mice, indicating that we are stimulating at the lower end of the cone sensitivity range. Thus, we may expect the recordable optomotor responses of transplanted *Aipl1*$^{-/-}$ animals to be improved with brighter stimulus light levels. An important question that remains to be determined is whether the new connections formed by transplanted photoreceptors and the host inner retinal neurons can faithfully reflect the spatiotemporal receptive fields of the intact retina. An indication that they may do comes from the optomotor data reported

significantly larger peak response amplitude, compared to *Aipl1*$^{-/-}$ + hCone-transplanted retinas at each tested irradiance (**p* < 0.05; *****p* < 0.001).

(E) Response latency decreased with increasing light intensity above 10^{12} photons/cm^2/s and was not significantly different between *Gnat1*$^{-/-}$ and *Aipl1*$^{-/-}$ + hCone-transplanted retinas at any irradiances where a measurable light response was observed (*p* > 0.05; two-Way ANOVA).

here, where acuity thresholds could be determined from transplanted eyes, while untreated animals showed no response.

Given the MEA responses and head-tracking behaviors, the lack of ERG responses in the same mice was disappointing but unsurprising. ERGs are averaged changes in polarization measured across the whole eye, while transplantations encompass a region of ~5 mm^2 (comparable to the size of the macula), compared with a surface area of ~50 mm^2. Moreover, ERG function is a fairly poor predictor of meaningful vision, with many patients presenting with little/no ERG but being capable of significant visually guided function (Berson, 2007).

Prior to this and our earlier study in the *Rd1* mouse (Ribeiro et al., 2021), we shared the concern of others as to whether inner retinal neurons retain sufficient plasticity to accept new photoreceptor inputs, and/or whether glial scarring might prove an impenetrable barrier, in advanced disease (Hippert et al., 2015, 2021). However, we now have promising evidence of the host inner retina being able to remodel and form new connections with human cones to bring about retinal and visual function within normal ambient light levels in two different models of advanced disease. Together, these support the notion that photoreceptor replacement therapy is a viable, potentially disease-agnostic, strategy for the treatment of advanced retinal degeneration.

METHODS

Detailed methods can be found in the supplemental information.

Animals

Aipl1$^{-/-}$, *Aipl1*$^{-/-}$/*FoxN1*$^{nu/nu}$ (generated in-house), *Gnat1*$^{-/-}$, and *C57Bl/6* (Charles River) WT animals were maintained in accordance with the UK Animals (Scientific Procedure) Act of 1986 and Policies on the Use of Animals and Humans in Neuroscience Research.

Human embryonic stem cells

The human H9 embryonic stem cell (ESC) line (WA09, female, ID/registry: WAe009-A (hPSCreg); Lot RB66492, P30) was acquired from WiCell and used in accordance with the International Society for Stem Cell Research (ISSCR) Standards for Human Stem Cell Use in Research.

Retinal differentiation culture and photoreceptor transplantation

ESCs were differentiated into retinal organoids and transduced with *ShH10.2.1L/M-Opsin.GFP* virus, and GFP+ hCones were isolated at 17–21 weeks of differentiation. 500,000 hCones/2μL were transplanted subretinally under direct visual control, as previously described (Gonzalez-Cordero et al., 2017; Ribeiro et al., 2021).

Histology and immunohistochemistry

Immunohistochemistry was performed as previously described (Ribeiro et al., 2021) with some modifications. Antibodies and staining conditions are provided in Table S1. Samples were imaged using a Zeiss LSM900 confocal microscope. Images shown are Maximum Intensity Projection images (MIPs) of xyz stacks (1 μm z-intervals, unless otherwise stated) or single xy images. Zeiss LSM image software, Imaris, ImageJ, and Adobe Photoshop were used for image processing.

Cell quantification

Images were taken of ×4 ROIs from superior mid/central retina, from 3 sections/retina from region containing the optic nerve (Figure S7). Nuclear size was determined by selecting cells at random and measuring the longest axis using ImageJ.

Retinal and visual function tests

Optomotor response

10 weeks post-transplantation, optokinetic reflexes were assessed using OptoDrum (Striatech, Tübingen, Germany). A striped pattern rotated at 12°/second, and visual acuity was tested at 99.72% contrast. Maximum irradiance at the platform was 8.8×10^{13} photons cm^2/s^1. Optokinetic responses were automatically detected in an unbiased manner. Each eye was tested on 3–4 separate occasions within 1 week. All individual test outcomes from all animals assessed are shown. $N = 15$ *Aipl1*$^{-/-}$ treated eyes, $N = 9$ *Aipl1*$^{-/-}$ untreated eyes, $N = 6$ *Gnat1*$^{-/-}$ eyes, and $N = 6$ *C57Bl/6* eyes.

Electroretinogram recordings

Dark-adapted ERG recordings were made using a Celeris ERG system with a full-field stimulator (Diagnosys, Massachusetts, USA). Eyes were recorded sequentially with the non-recorded eye acting as the reference electrode. Dark-adapted, single-flash recordings were obtained at a range of light intensities. 10 responses were averaged for light intensities of 0.001, 0.01, 0.1, and 1 cd s/m^2, and 5 responses for 10 and 30 cd s/m^2; bandpass filter was set between 0.125 and 300 Hz.

Multi-electode Array recordings

MEA recordings and analysis were performed as previously described (see supplemental methods). In brief, *Gnat1*$^{-/-}$ ($N = 4$), human cone-transplanted *Aipl1*$^{-/-}$ ($N = 4$), and untreated *Aipl1*$^{-/-}$ ($N = 3$) mice were euthanized by cervical dislocation ~3 months post-transplantation. Eyes were

enucleated, and retinal isolation was performed in the dark in warm carboxygenated Ames' media supplemented with 1.9 g/L sodium bicarbonate (Sigma-Aldrich, UK). The retina was incised into a Maltese cross motif and mounted onto a perforated MEA (120pMEA100/30iR-ITO; Multi Channel Systems, Reutlingen, Germany) with the GCL facing down onto the electrodes. For transplanted animals, GFP+ regions of the retinal cell mass were placed centrally over the electrodes to maximize the recording area covered by transplanted hCones.

Spike sorting

Neural waveforms were processed using Offline Sorter (v.4.7.1; Plexon). Single-unit data were subsequently sent to and stored in NeuroExplorer (v.5.437; Nex Technologies) and analyzed by custom written MATLAB codes, and neuronal responses were classified as described in Ribeiro et al., 2021.

Statistical analysis

All values are presented mean ± SD (standard deviation) unless otherwise stated; N, number of animals, retinas, or independent experiments performed; n, number of cells, images examined, or single units. Statistical significance was assessed using GraphPad Prism software and denoted as $p < 0.05$ = *; $p < 0.01$ = **; $p < 0.001$ = ***. Appropriate statistical tests were applied including 2 tailed t test (Mann-Whitney), one-way ANOVA with Tukey's correction for multiple comparisons, two-way ANOVA with Bonferroni's correction, and paired/unpaired t tests.

RESOURCE AVAILABILITY

Lead contact

Requests for further information and resources should be directed to and will be fulfilled by the lead contact, Rachael A. Pearson.

Materials availability

All unique/stable reagents generated in this study will be made available on request but may require payment and/or completed Materials Transfer Agreement if there is potential for commercial application. *Aipl1*$^{-/-}$/*Foxn1*^{nu} mice will be provided directly; in the event of high demand, and presuming the strain will be accepted, the lead contact will deposit the strain with Jackson Laboratory.

Data and code availability

Datasets supporting the current study have not been deposited in a public repository; the authors are undertaking further analysis, and any outputs arising from these will be published in due course but are available on reasonable request. The codes used have been reported previously and will be shared by the lead contacts upon request.

ACKNOWLEDGMENTS

This work was supported by grants from the Medical Research Council UK (MR/T002735/2, MR/V038559/1) and an unrestricted award from Guy's and St Thomas's Trust. A.M. is funded by the Wellcome Trust Advanced Therapies for Regenerative Medicine PhD Program, 218461/Z/19/Z). This work was made possible by the dedication and skills of the Ocular Cell and Gene Therapy group, with particular thanks to Dr M. Khazim, Dr P. Harding, B. Ladino, E. Lanning, M. Margari, C. Mofidi, K. Kumar, S. Guilfoyle, and S. van Heerden for help with stem cell maintenance cultures; I. Mahamoud for viral vector production; and Guy's Clinical Trials Unit cell sorting facility. The authors have applied a Creative Commons Attribution (CC BY) license to any Author Accepted Manuscript version arising. Figure 2P–2R schematics were generated using BioRender (https://biorender.com/).

AUTHOR CONTRIBUTIONS

Conceptualization: C.A.P., A.G.-C., R.R.A., and R.A.P.; methodology: C.A.P., A.M., J.L., J.D.D., J.R., M.T., M.J.B., E.L.W., M.M.K., A.J.S., A.G.-C., R.R.A., and R.A.P.; preliminary investigation: C.A.P., J.R., A.G.-C., and R.A.P.; investigation: C.A.P., A.M., J.L., J.D.D., J.R., J.K., M.J.B., E.L.W., M.T., A.G.-C., and R.A.P.; analysis and interpretation: C.A.P., A.M., J.L., M.T., A.J.S., R.R.A., and R.A.P.; writing – original draft: J.R., R.A.P., and C.A.P.; writing – review and editing: C.A.P., A.M., A.J.S., R.R.A., and R.A.P.; funding acquisition: R.R.A. and R.A.P.; supervision: R.R.A. and R.A.P.

DECLARATION OF INTERESTS

The authors declare no competing interests.

SUPPLEMENTAL INFORMATION

Supplemental information can be found online at https://doi.org/10.1016/j.stemcr.2025.102470.

Received: December 18, 2024
Revised: March 3, 2025
Accepted: March 3, 2025
Published: March 27, 2025

REFERENCES

Akiba, R., Lind Boniec, S., Knecht, S., Uyama, H., Tu, H.Y., Baba, T., Takahashi, M., Mandai, M., and Wong, R.O. (2024). Cellular and circuit remodeling of the primate foveal midget pathway after acute photoreceptor loss. Proc. Natl. Acad. Sci. USA *121*, e2413104121. https://doi.org/10.1073/pnas.2413104121.

Akiba, R., Tu, H.Y., Hashiguchi, T., Takahashi, Y., Toyooka, K., Tsukamoto, Y., Baba, T., Takahashi, M., and Mandai, M. (2024). Host-Graft Synapses Form Functional Microstructures and Shape the Host Light Responses After Stem Cell-Derived Retinal Sheet Transplantation. Investig. Ophthalmol. Vis. Sci. *65*, 8. https://doi.org/10.1167/iovs.65.12.8.

Barber, A.C., Hippert, C., Duran, Y., West, E.L., Bainbridge, J.W.B., Warre-Cornish, K., Luhmann, U.F.O., Lakowski, J., Sowden, J.C., Ali, R.R., and Pearson, R.A. (2013). Repair of the degenerate retina by photoreceptor transplantation. Proc. Natl. Acad. Sci. USA *110*, 354–359. https://doi.org/10.1073/pnas.1212677110.

Beier, C., Hovhannisyan, A., Weiser, S., Kung, J., Lee, S., Lee, D.Y., Huie, P., Dalal, R., Palanker, D., and Sher, A. (2017). Deafferented

Adult Rod Bipolar Cells Create New Synapses with Photoreceptors to Restore Vision. J. Neurosci. *37*, 4635–4644. https://doi.org/10.1523/JNEUROSCI.2570-16.2017.

Berson, E.L. (2007). Long-term visual prognoses in patients with retinitis pigmentosa: the Ludwig von Sallmann lecture. Exp. Eye Res. *85*, 7–14. https://doi.org/10.1016/j.exer.2007.03.001.

Calvert, P.D., Krasnoperova, N.V., Lyubarsky, A.L., Isayama, T., Nicoló, M., Kosaras, B., Wong, G., Gannon, K.S., Margolskee, R.F., Sidman, R.L., et al. (2000). Phototransduction in transgenic mice after targeted deletion of the rod transducin alpha -subunit. Proc. Natl. Acad. Sci. USA *97*, 13913–13918. https://doi.org/10.1073/pnas.250478897.

D'Orazi, F.D., Suzuki, S.C., and Wong, R.O. (2014). Neuronal remodeling in retinal circuit assembly, disassembly, and reassembly. Trends Neurosci. *37*, 594–603. https://doi.org/10.1016/j.tins.2014.07.009.

Gonzalez-Cordero, A., Goh, D., Kruczek, K., Naeem, A., Fernando, M., Kleine Holthaus, S.M., Takaaki, M., Blackford, S.J.I., Kloc, M., Agundez, L., et al. (2018). Assessment of AAV Vector Tropisms for Mouse and Human Pluripotent Stem Cell-Derived RPE and Photoreceptor Cells. Hum. Gene Ther. *29*, 1124–1139. https://doi.org/10.1089/hum.2018.027.

Gonzalez-Cordero, A., Kruczek, K., Naeem, A., Fernando, M., Kloc, M., Ribeiro, J., Goh, D., Duran, Y., Blackford, S.J.I., Abelleira-Hervas, L., et al. (2017). Recapitulation of Human Retinal Development from Human Pluripotent Stem Cells Generates Transplantable Populations of Cone Photoreceptors. Stem Cell Rep. *9*, 820–837. https://doi.org/10.1016/j.stemcr.2017.07.022.

Hippert, C., Graca, A.B., Barber, A.C., West, E.L., Smith, A.J., Ali, R.R., and Pearson, R.A. (2015). Muller glia activation in response to inherited retinal degeneration is highly varied and disease-specific. PLoS One *10*, e0120415. https://doi.org/10.1371/journal.pone.0120415.

Hippert, C., Graca, A.B., Basche, M., Kalargyrou, A.A., Georgiadis, A., Ribeiro, J., Matsuyama, A., Aghaizu, N., Bainbridge, J.W., Smith, A.J., et al. (2021). RNAi-mediated suppression of vimentin or glial fibrillary acidic protein prevents the establishment of Muller glial cell hypertrophy in progressive retinal degeneration. Glia *69*, 2272–2290. https://doi.org/10.1002/glia.24034.

Klymenko, V., González Martínez, O.G., and Zarbin, M.A. (2024). Recent Progress in Photoreceptor Cell-Based Therapy for Degenerative Retinal Disease. Stem Cells Transl. Med. *13*, 332–345. https://doi.org/10.1093/stcltm/szae005.

Marc, R.E., Jones, B.W., Watt, C.B., and Strettoi, E. (2003). Neural remodeling in retinal degeneration. Prog. Retin. Eye Res. *22*, 607–655. https://doi.org/10.1016/s1350-9462(03)00039-9.

Matsuyama, A., Kalargyrou, A.A., Smith, A.J., Ali, R.R., and Pearson, R.A. (2022). A comprehensive atlas of Aggrecan, Versican, Neurocan and Phosphacan expression across time in wildtype retina and in retinal degeneration. Sci. Rep. *12*, 7282. https://doi.org/10.1038/s41598-022-11204-w.

Nishiguchi, K.M., Carvalho, L.S., Rizzi, M., Powell, K., Holthaus, S.M.k., Azam, S.A., Duran, Y., Ribeiro, J., Luhmann, U.F.O., Bainbridge, J.W.B., et al. (2015). Gene therapy restores vision in rd1 mice after removal of a confounding mutation in Gpr179. Nat. Commun. *6*, 6006. https://doi.org/10.1038/ncomms7006.

Parnami, K., and Bhattacharyya, A. (2023). Current approaches to vision restoration using optogenetic therapy. Front. Cell. Neurosci. *17*, 1236826. https://doi.org/10.3389/fncel.2023.1236826.

Pearson, R.A., Barber, A.C., Rizzi, M., Hippert, C., Xue, T., West, E.L., Duran, Y., Smith, A.J., Chuang, J.Z., Azam, S.A., et al. (2012). Restoration of vision after transplantation of photoreceptors. Nature *485*, 99–103. https://doi.org/10.1038/nature10997.

Pearson, R.A., Gonzalez-Cordero, A., West, E.L., Ribeiro, J.R., Aghaizu, N., Goh, D., Sampson, R.D., Georgiadis, A., Waldron, P.V., Duran, Y., et al. (2016). Donor and host photoreceptors engage in material transfer following transplantation of post-mitotic photoreceptor precursors. Nat. Commun. *7*, 13029. https://doi.org/10.1038/ncomms13029.

Procyk, C.A., Eleftheriou, C.G., Storchi, R., Allen, A.E., Milosavljevic, N., Brown, T.M., and Lucas, R.J. (2015). Spatial receptive fields in the retina and dorsal lateral geniculate nucleus of mice lacking rods and cones. J. Neurophysiol. *114*, 1321–1330. https://doi.org/10.1152/jn.00368.2015.

Puthussery, T., Gayet-Primo, J., and Taylor, W.R. (2010). Localization of the calcium-binding protein secretagogin in cone bipolar cells of the mammalian retina. J. Comp. Neurol. *518*, 513–525. https://doi.org/10.1002/cne.22234.

Ramamurthy, V., Niemi, G.A., Reh, T.A., and Hurley, J.B. (2004). Leber congenital amaurosis linked to AIPL1: a mouse model reveals destabilization of cGMP phosphodiesterase. Proc. Natl. Acad. Sci. USA *101*, 13897–13902. https://doi.org/10.1073/pnas.0404197101.

Ribeiro, J., Procyk, C.A., West, E.L., O'Hara-Wright, M., Martins, M.F., Khorasani, M.M., Hare, A., Basche, M., Fernando, M., Goh, D., et al. (2021). Restoration of visual function in advanced disease after transplantation of purified human pluripotent stem cell-derived cone photoreceptors. Cell Rep. *35*, 109022. https://doi.org/10.1016/j.celrep.2021.109022.

Scalabrino, M.L., Thapa, M., Wang, T., Sampath, A.P., Chen, J., and Field, G.D. (2023). Late gene therapy limits the restoration of retinal function in a mouse model of retinitis pigmentosa. Nat. Commun. *14*, 8256. https://doi.org/10.1038/s41467-023-44063-8.

Singh, R.K., Kolandaivelu, S., and Ramamurthy, V. (2014). Early alteration of retinal neurons in Aipl1-/- animals. Investig. Ophthalmol. Vis. Sci. *55*, 3081–3092. https://doi.org/10.1167/iovs.13-13728.

Strettoi, E., Pignatelli, V., Rossi, C., Porciatti, V., and Falsini, B. (2003). Remodeling of second-order neurons in the retina of rd/rd mutant mice. Vis. Res. *43*, 867–877. https://doi.org/10.1016/s0042-6989(02)00594-1.

Tan, M.H., Mackay, D.S., Cowing, J., Tran, H.V., Smith, A.J., Wright, G.A., Dev-Borman, A., Henderson, R.H., Moradi, P., Russell-Eggitt, I., et al. (2012). Leber congenital amaurosis associated with

AIPL1: challenges in ascribing disease causation, clinical findings, and implications for gene therapy. PLoS One *7*, e32330. https://doi.org/10.1371/journal.pone.0032330.

Wong, K.Y., Dunn, F.A., Graham, D.M., and Berson, D.M. (2007). Synaptic influences on rat ganglion-cell photoreceptors. J. Physiol. *582*, 279–296. https://doi.org/10.1113/jphysiol.2007.133751.

Zhao, X., Stafford, B.K., Godin, A.L., King, W.M., and Wong, K.Y. (2014). Photoresponse diversity among the five types of intrinsically photosensitive retinal ganglion cells. J. Physiol. *592*, 1619–1636. https://doi.org/10.1113/jphysiol.2013.262782.

Stem Cell Reports, Volume *20*

Supplemental Information

Human cone photoreceptor transplantation stimulates remodeling and restores function in AIPL1 model of end-stage Leber congenital amaurosis

Christopher A. Procyk, Anna Melati, Joana Ribeiro, Jingshu Liu, Matthew J. Branch, Jamie D. Delicata, Menahil Tariq, Aikaterini A. Kalarygrou, Jessica Kapadia, Majid Moshtagh Khorsani, Emma L. West, Alexander J. Smith, Anai Gonzalez-Cordero, Robin R. Ali, and Rachael A. Pearson

SUPPLEMENTAL INFORMATION

Contains: Supplemental Figures S1 – S7 and figure legends

Supplemental Experimental Methods

SUPPLEMENTAL FIGURES

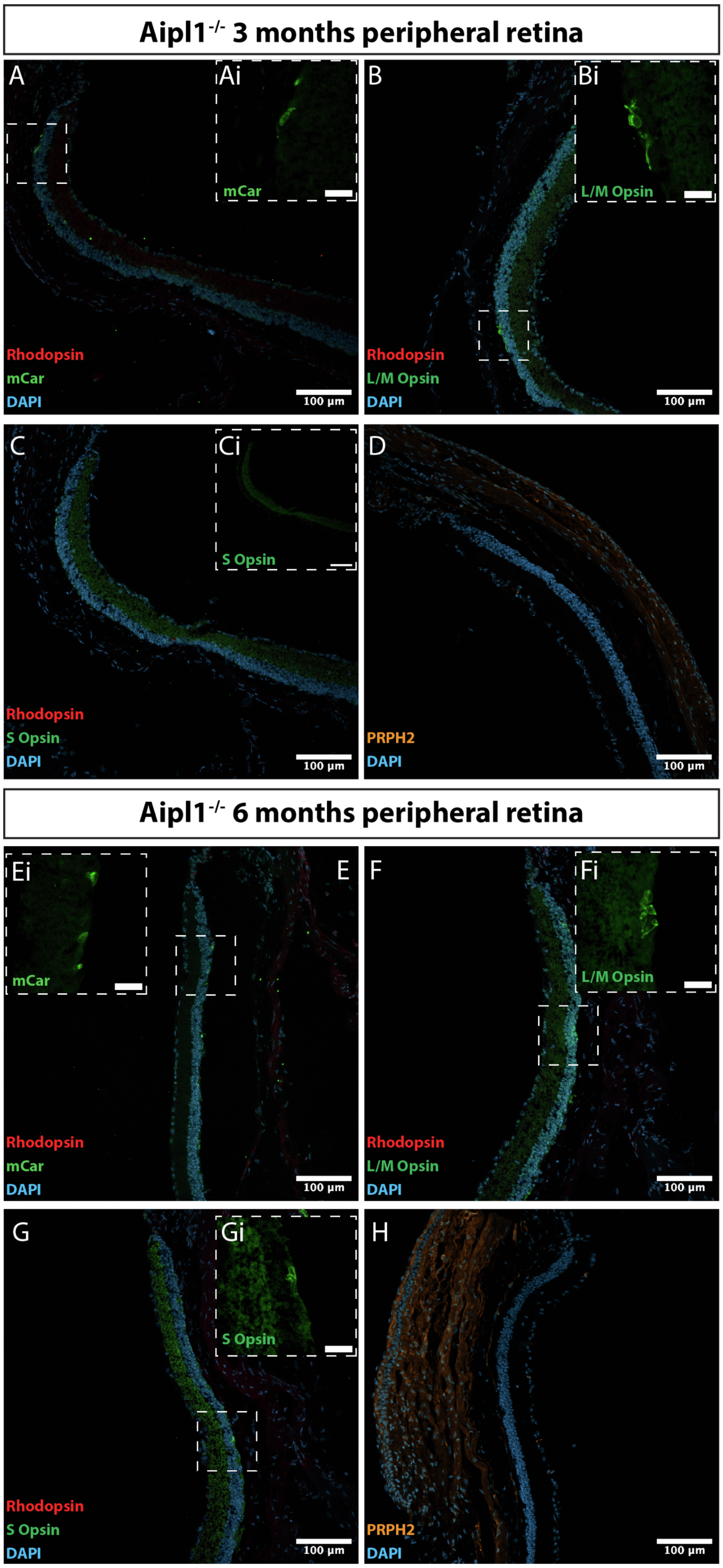

Supplemental Figure S1. Photoreceptor degeneration in the periphery of *Aipl1*$^{-/-}$ retina

(A-D) Periphery of representative 3-month-old *Aipl1*$^{-/-}$ retinae immunolabelled for rod (Rhodopsin, *red*) and cone (mCar, M/L opsin, S Opsin, *green*) photoreceptor markers. No rhodopsin+ rods were seen at 3 months of age in the *Aipl1*$^{-/-}$ peripheral retina. Occasional mCar+ and L/M opsin+ cones were seen, but S opsin was not detected **(Ai, Bi, Ci)**. No labelling for the outer segment protein Prph2+ was seen **(D)**. **(E-H)** At 6 months of age the ONL of *Aipl1*$^{-/-}$ mice is completely degenerated, with only very sporadic cones seen in the periphery **(Ei, Fi, Gi)**. No Prph2+ labelling was visible **(H)**. Scale bar: A-H, 50µm; Ai, Bi, Ci, Ei, Fi, Gi, 20µm.

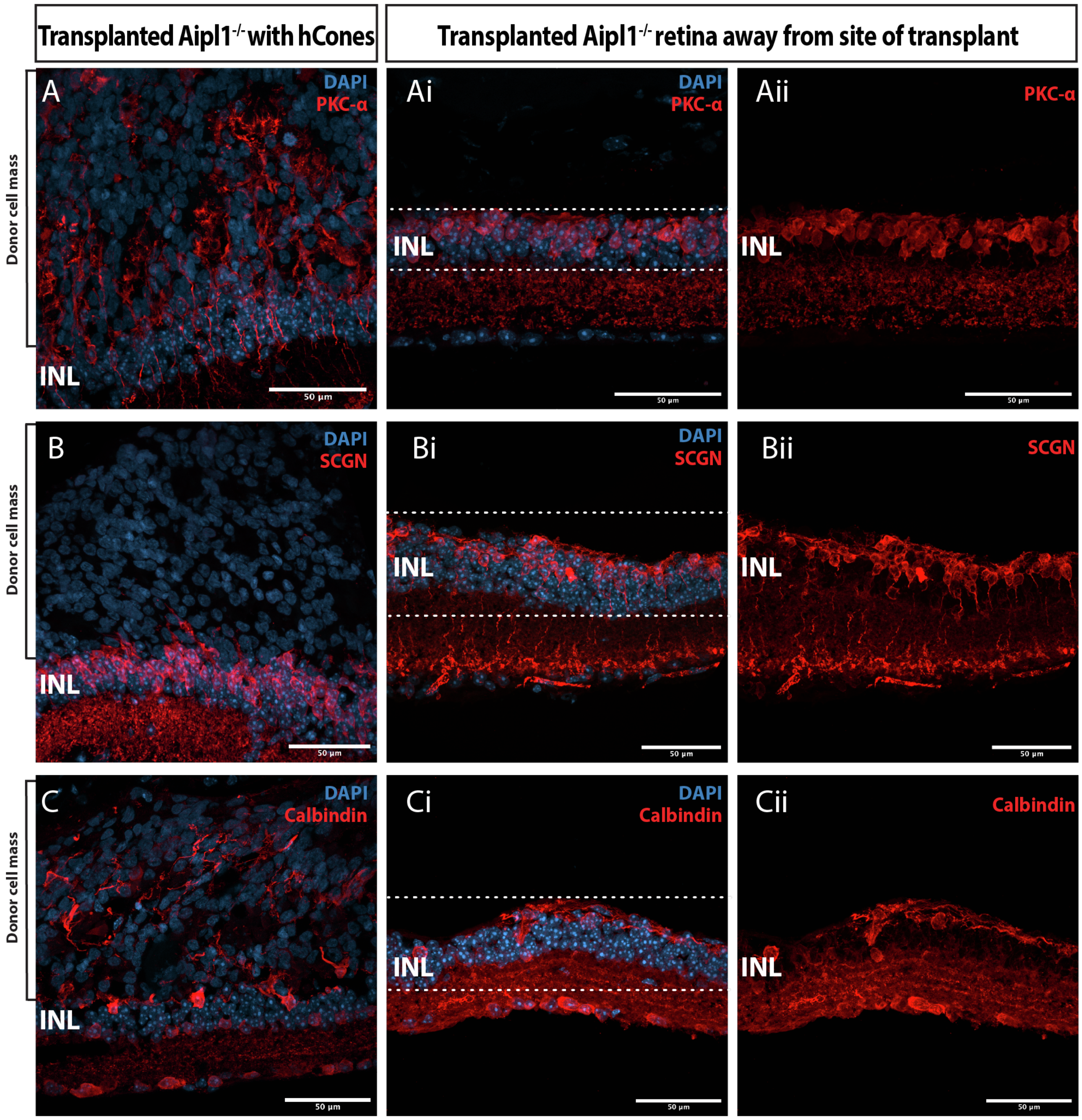

Supplemental Figure S2: INL undergoes extensive remodelling in regions immediately beneath transplanted with human cones. (A-C) Different regions in the same retinae with or without hCone donor cell mass show markedly different extents of neural remodelling. **(A-Aii)** PKC-α+ ON BCs show significant dendritic extension up into the hCone donor cell mass, while in the same retina away from the engraftment site, BCs show no neurite remodelling. **(B-Bii)** SCGN staining for cone BCs shows a similar pattern. (**B**) cone BCs show clear neurite extension toward the donor cell mass, while cone BCs located distant to the donor cell mass extend their dendrites horizontally, similar to age-matched

Aipl1$^{-/-}$ degenerated retina. **(C-Cii)** HCs, immunolabelled for calbindin, exhibit the same pattern of remodelling, those under the donor cell mass extending neurites towards it, while those distant to the donor cell mass remain unchanged from control *Aipl1*$^{-/-}$. Scale bar 50μm. INL – inner nuclear layer. Dapi – nuclear label.

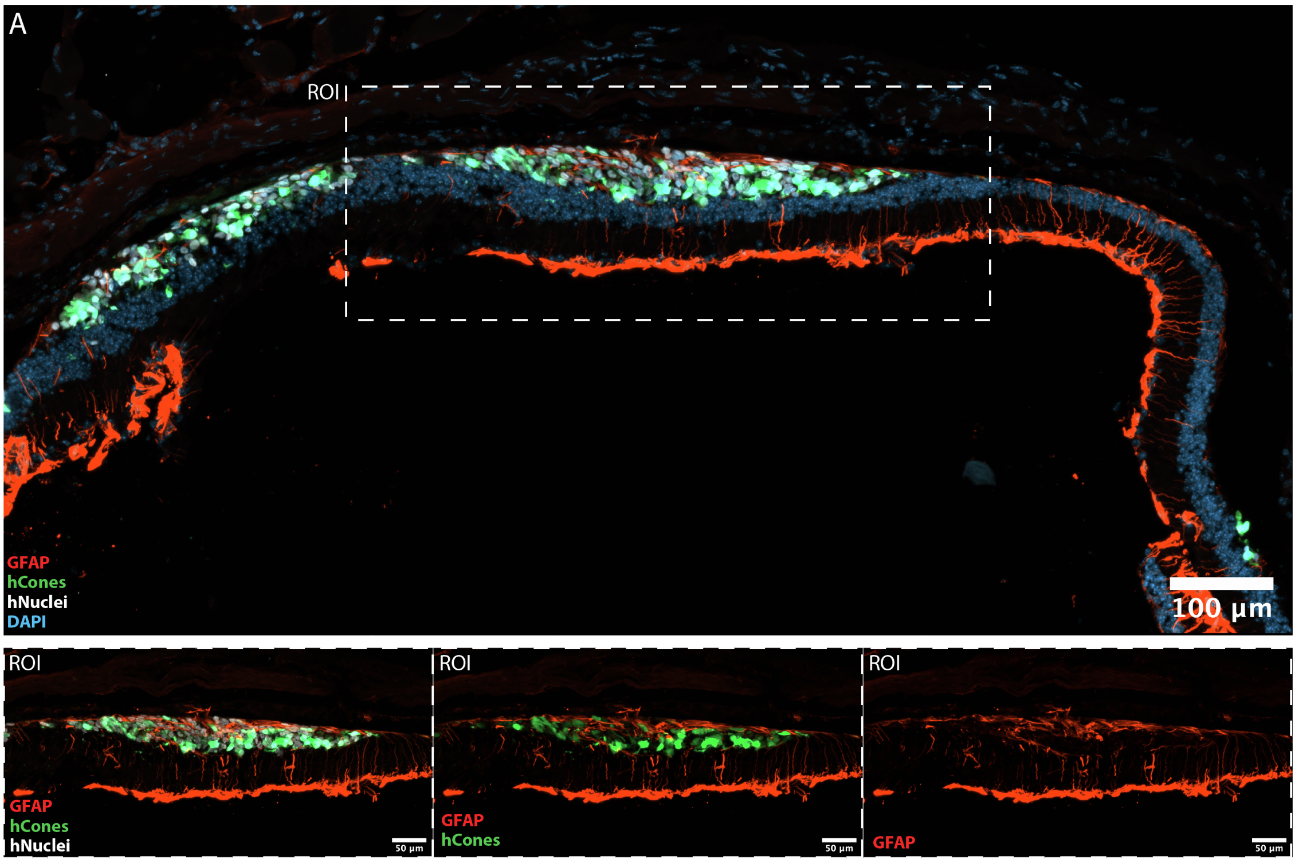

Supplemental Figure S3: Muller glia cells within the recipient retina extend into and delineate the outer edge of the human cone graft. (A) In regions of the retina with GFP+ hCones (*green*), Müller glial cells are activated, and upregulate Gfap (*red*). The processes of Müller glia cells extend from the ganglion cell layer through the host retina towards the hCone donor cell mass. HNA (hNuclei) colocalises with GFP+ hCones but not Gfap+ Müller glia, indicating that the Müller glia predominantly derive from the host mouse retina. **ROIs** show glial cell processes extending up through the graft, delineating its apical margin thereby incorporating the graft within the host retinal structure. Main image is a confocal maximum image projection tile scan (digital stitching), ROIs are digital zooms from the same tile scan. Scale bar 100 µm, 50 µm for ROIs.

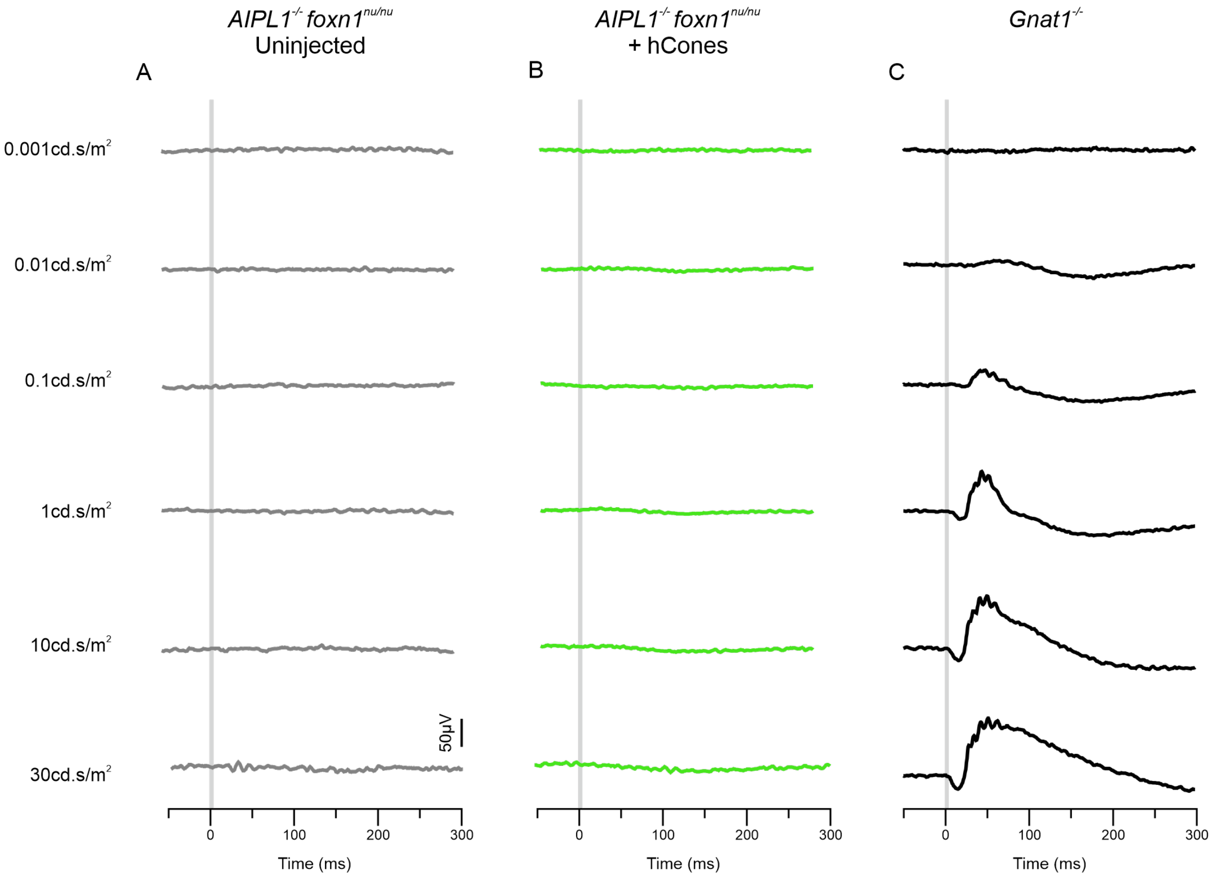

Supplemental Figure S4: Transplanted human cones do not drive reproducible ERG responses in the *Aipl1*$^{-/-}$ + human cones treated retina

A-C, Mean dark-adapted ERG traces from all animals recorded at 6 months age in (**A**) uninjected *Aipl1*$^{-/-}$ mice (N = 3), **(B)** *Aipl1*$^{-/-}$ + hCone transplanted mice, 3 months post-transplantation (N = 4) and **(C),** age-matched *Gnat1*$^{-/-}$ mice (N = 4), which exhibit cone-only mediated function and serve as a positive control. A small response was observed in *Gnat1*$^{-/-}$ animals from 0.01 cd.s/m^2, with measurable a- and b-waves from 0.1 cd.s/m^2 but no reproducible response was seen in either the un-injected or the transplanted *Aipl1*$^{-/-}$ mice at any intensity.

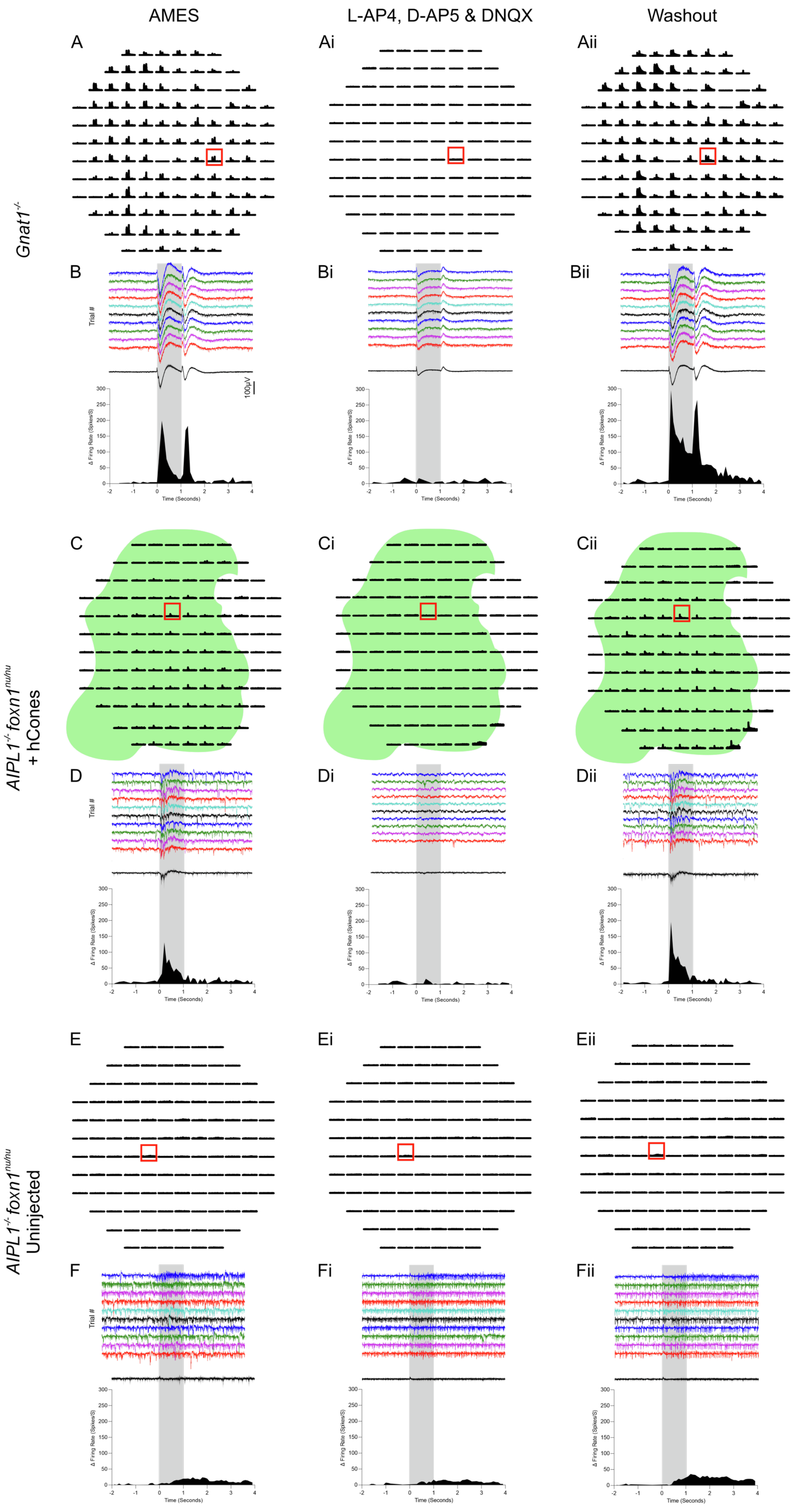

AMES
L-AP4, D-AP5 & DNQX
Washout
A
Ai
Aii
Gnat1-/-
B
Bi
Bii
Trial #
100μV
Δ Firing Rate (Spikes/S)
Time (Seconds)
C
Ci
Cii
AIPL1-/- foxn1nu/nu + hCones
D
Di
Dii
E
Ei
Eii
AIPL1-/- foxn1nu/nu Uninjected
F
Fi
Fii

Supplemental Figure S5: Transplanted human cones connect to the host *Aipl1*$^{-/-}$ retina through functional glutamatergic synapses in the outer retina

Multi-unit spiking activity before, during, and after synaptic blockade. (**A**) Representative *Gnat1*$^{-/-}$ retina. Transient increases and decreases in firing rate were observed following light onset and/or offset. (**Ai & Aii)** These were reversibly abolished by synaptic blockers. (**B-Bii**) mERG and multi-unit PSTH (magnified from the *red box* in **A**) shows fast cone-driven response, which is reversibly abolished by pharmacological intervention. (**C**) Representative *Aipl1*$^{-/-}$+ hCone transplanted retina. Transient increases in firing rate correlated with the position of overlying GFP+ hCones following stimulation with a 1s light pulse. (**Ci & Cii)** Addition of synaptic blockers reversibly abolished these responses. (**D-Dii**) Light-evoked mERG and multi-unit PSTH (*red box* in **C**) illustrates hCone-driven light responses that are both abolished by pharmacological intervention and return following washout. (**E**) Representative untreated age-matched *Aipl1*$^{-/-}$ retina. Most channels were not light responsive and (**Ei & Eii)** there was no effect of synaptic blockers on these few light responsive channels. (**F-Fii**) No discernible mERGs were seen on the few channels which demonstrated a deafferented ipRGC responses following light onset (*red box*), which were not eradicated by synaptic blockers, as expected for deafferented intrinsically photosensitive retinal ganglion cell responses.[30]

Scale bars: 400 Spikes/s, 5 s (A, C, and E); Green overlay indicates region of GFP+ cell mass. Grey bars in B, D & F indicate duration of light pulse.

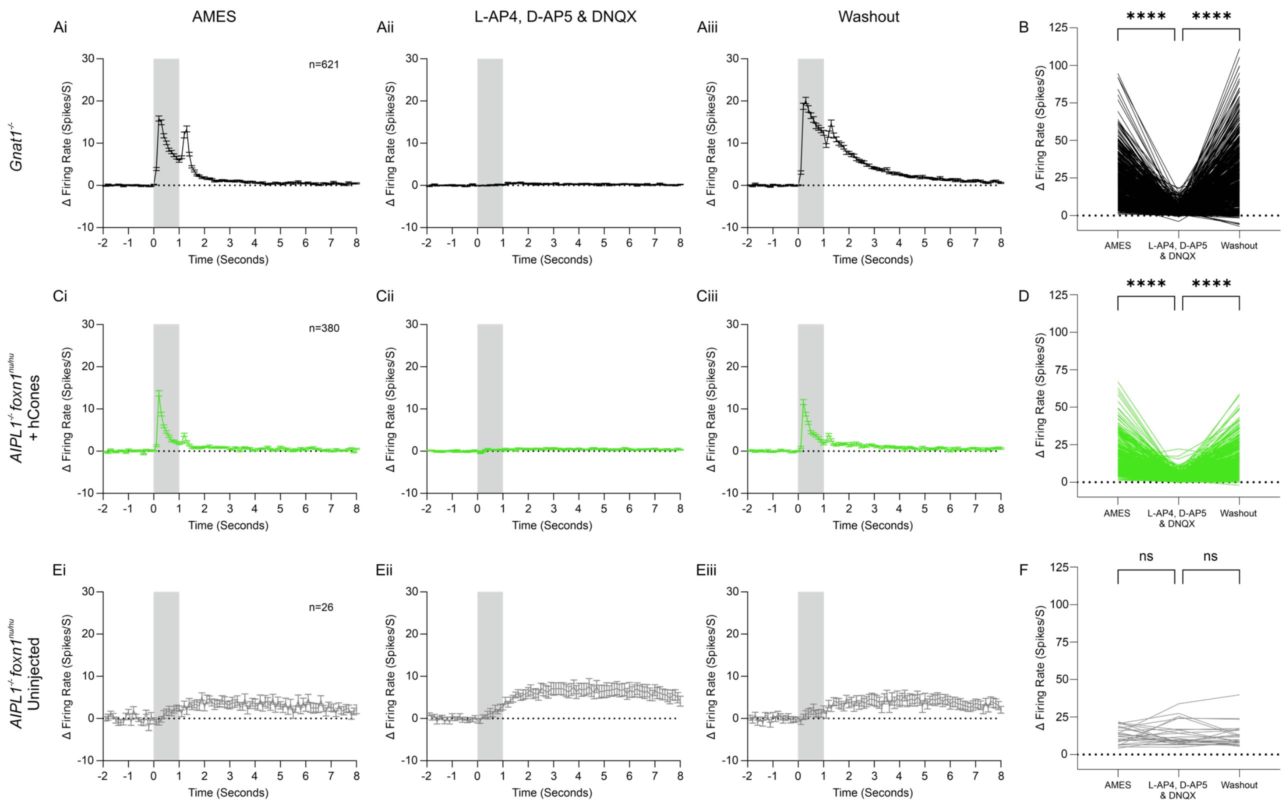

Supplemental Figure S6: Single unit quantification of Glutamatergic transmission of visual information at the human cone-host bipolar cell synapse.

(**A**) Average PSTH (Mean ±SEM) of all ON-type responses in *Gnat1*$^{-/-}$ retinas (n=621 units) demonstrates a robust light response when stimulated with a 1s light pulse from darkness (*left*), which is eradicated during application of synaptic blockers (middle) and returns following Washout (*right*). (**B**) Peak ON response amplitude of all single units in A demonstrates a significant reduction in amplitude under synaptic blockade (Mean ±SEM; 2.75 ± 0.13 Spikes/s) when compared to the AMES (22.42 ± 0.6 Spikes/s) and Washout conditions (25.95 ± 0.84 Spikes/s; 2-Way Anova; $p < 0.001$). (**C**) Average PSTH of ON-type responses in *Aipl1*$^{-/-}$ + human cone transplanted retinas (n = 380) show a robust light response to the same 1s light pulse from darkness, (*left*) which is eradicated during synaptic blockade (middle) and returns upon Washout (*right)*. (**D**) Peak ON response Amplitude of all single units in C demonstrates a significant reduction in amplitude under synaptic blockade (3.21± 0.14 Spikes/s) when compared to the AMES (16.79 ± 0.56 Spikes/s) and Washout conditions (14.95 ± 0.56 Spikes/s; 2-Way Anova; $p < 0.001$). **(E)** Average PSTH of all ON-type responses in untreated *Aipl1*$^{-/-}$ retinas (n= 26 units) demonstrates slow and sustained light responses originated from intrinsically photosensitive retinal ganglion cells

when stimulated with a 1s light pulse from darkness (*left*), which is not eradicated during application of synaptic blockers (*middle*) and remains following Washout (*right*). **(F)** Peak response amplitude is not significantly different under synaptic blockade (13.32 ± 1.56 Spikes/s) when compared to AMES (12.63 ± 1.06 Spikes/s; 2 Way Anova, $p = 0.85$) or Washout conditions (12.38 ± 1.53 Spikes/s; 2-Way Anova; $p = 0.74$)

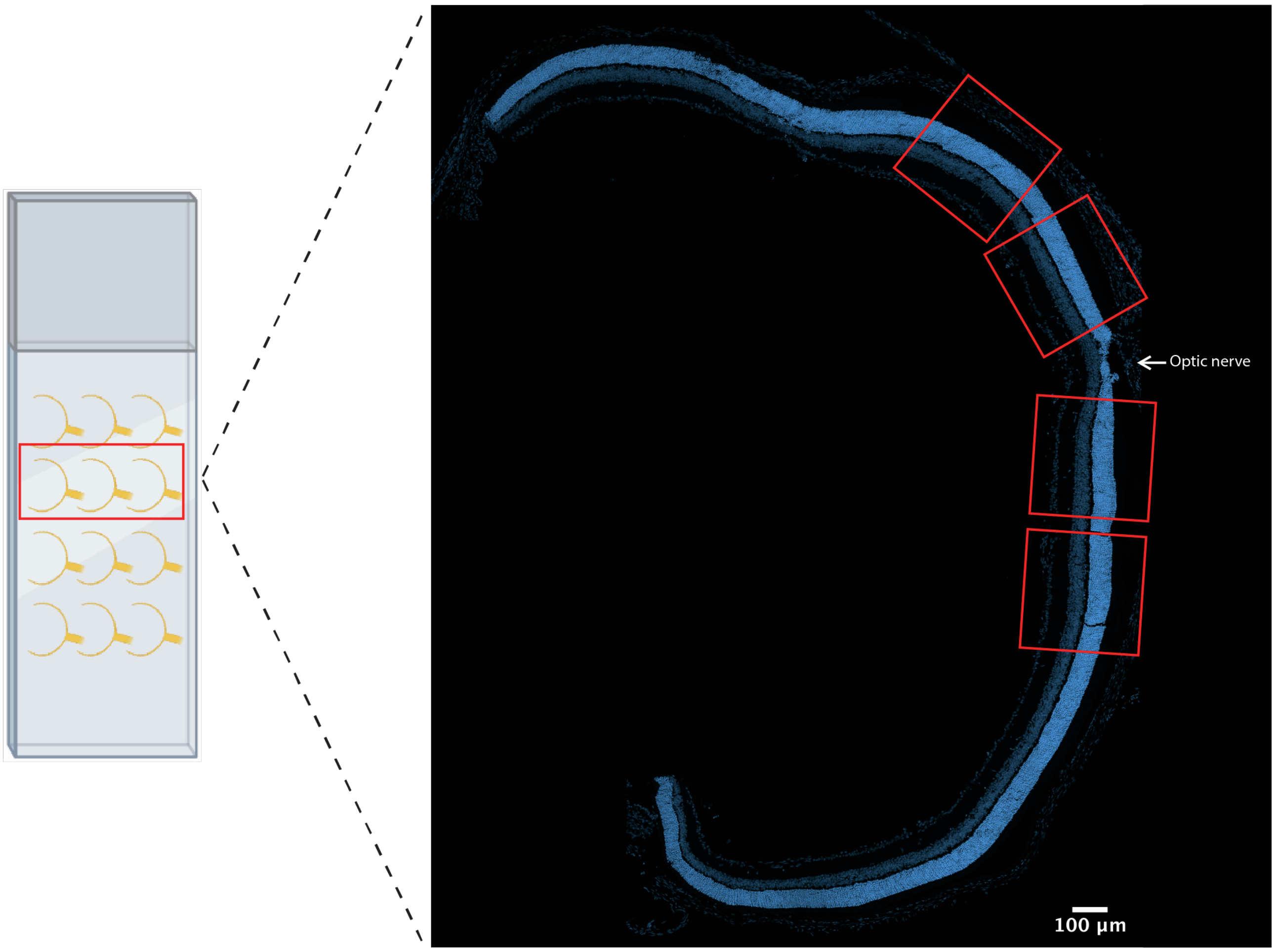

Supplemental Figure S7. Schematic and confocal tile scan (digital stitching) image showing the locations of the four regions (*red boxes*) selected for imaging of immunohistological assessments of inner retinal remodelling.

SUPPLEMENTARY EXPERIMENTAL METHODS

EXPERIMENTAL PROCEDURES

Animals

Aipl1$^{-/-}$, *Aipl1*$^{-/-}$/*FoxN1*$^{nu/nu}$ (line generated in-house), *Gnat1*$^{-/-}$ (Calvert *et al*., 2000), and *C57Bl/6* (Charles River) animals were maintained on a standard 12 hr light-dark cycle. Mice received food and water *ad libitum* and were provided with fresh bedding and nesting daily. Male and female mice were used without discrimination for experiments and are represented in approximately equal numbers. All experiments have been conducted in accordance with the United Kingdom Animals (Scientific Procedure) Act of 1986 and Policies on the Use of Animals and Humans in Neuroscience Research and ARVO Statement for the Use of Animals in Ophthalmic and Vision Research.

hESC maintenance culture

The human H9 ESC line (WA09, female, ID/registry: WAe009-A (hPSCreg); Lot RB66492, P30) was acquired directly from WiCell and used in accordance with the ISSCR Standards for Human Stem Cell Use in Research. In brief, a working cell bank (WCB) was cryopreserved at P33 in Knockout™ Serum Replacement (#10828010, ThermoFisher) with 10% CryoSure DMSO (#WAK-DMSO-10, WAK). These cells were fully characterised, including Karyometrix analysis (Stemnovate) with no major chromosomal changes detected (>60bps). The undifferentiated state of the cell population was assessed by flow cytometry (Human Pluripotent SC Analysis kit, #560461 and #560589, BD Biosciences) with all pluripotent markers present in >70% of cells and differentiation markers present in <10% of cells. Each experimental run initiated from a freshly thawed vial of the WCB, with cells seeded for differentiation within 15 passages. ESCs were maintained under feeder-free conditions in Essential 8™ media (#A1517001; ThermoFisher) on Geltrex™ (#A1413301;

ThermoFisher) coated 6 well plates. They were fed daily and grown to between 60-80% confluence, before passaging with Versene (#15040066; ThermoFisher) and seeded at a density of $2x10^4$ cells/cm^2. Routine mycoplasma (MycoAlert plus detection kit, #LT07-710, Lonza) and sterility testing (Tryptic Soy broth, #1463170010 and Fluid Thioglycollate media, #STBMFTM12, Merck) was performed monthly for all cell cultures.

Retinal differentiation culture and photoreceptor transplantation

ESCs were differentiated into retinal organoids (hROs), transduced with *ShH10.2.1L/MOpsin.GFP* virus, and GFP^{+ve} human cones were isolated at 17-21 wks of differentiation, as we have previously described (Gonzalez-Cordero et al., 2017; Ribeiro et al., 2021) with minor modifications. GFP+ purity was > 80% in all transplants (mean 81.0% ± 12.3, N = 4 sorts), as verified by flow cytometry post-sort. Sorted cells were re-suspended in EBSS at a concentration of 5 x 10^5 / 2μL and administered by subretinal injection. All physiological and histological assessments were performed between 10-14 weeks (~3 months) post-transplantation.

Surgery and transplantation

All surfaces were wiped with 70% ethanol prior to surgery. Mice were anesthetized by intraperitoneal injection (10 mL/Kg) of a mixture of a mixture of Dormitor (1 mg/ml medetomidine hydrochloride; Pfizer Pharmaceuticals), ketamine (100 mg/ml; Fort Dodge Animal Health), and sterile water in the ratio 5:3:42. Tropicamide (1 %; Bausch & Lomb) was used to dilate the pupils and topical anaesthetic was applied (Tetracaine). Eyes were kept moist by using Viscotears (Novartis Pharmaceuticals UK Ltd). Surgery was performed under direct visual control using an operating microscope (Zeiss). A sterile 34-gauge hypodermic needle was used to make a transcleral entry in the posterior orbit and slowly inject 2 μl of

cell suspension into the sub-retinal space. The same region of the eye was targeted for all injections. Significant care was taken not to rupture the very thin remaining neural retina. Anaesthesia was then reversed using same amount of Antisedan, 10 mL/Kg, (atipamezole hydrochloride 0.10 mg/ml, Pfizer Pharmaceuticals, Kent UK), with the mice placed on heat mats until fully recovered.

Histology and immunohistochemistry

Mice were euthanized by cervical dislocation 3 months post-transplantation, unless otherwise noted. Eyes were dissected to remove the cornea, iris, and lens. All samples were fixed in 4% PFA, cryopreserved in 20% sucrose (30 minutes at room temperature or overnight at 4°C) and embedded in OCT before samples were cryo-sectioned at 12 µm thickness across 6 slides and stored at –20°C. Immunohistochemistry was performed broadly as previously described (Ribeiro et al., 2021), with some modifications. A list of primary and secondary antibodies and staining conditions is provided in **Supplemental Table S1**. Samples were imaged using a Zeiss LSM900 confocal microscope. Images shown are maximum projection images (MIPs) of xyz stacks, at 1µm intervals in the z plane, unless otherwise stated (whereupon single section images may be shown to improve clarity and/or confirm co-localization). Zeiss LSM image software, Imaris, ImageJ and Adobe Photoshop were used for image processing.

Cell quantification

To quantify PKC-α, Secretagogin and HuC/D positive cells, confocal images (162.5 x 162.5 µm) were taken of x4 Regions of Interest (ROI) from the superior mid/central retina, from 3 sections per retina from the region containing the optic nerve (**Supplementary Figure S7**). Counts were made at 3 months and 6 months of age, or as detailed in the Results. All counts

included age matched wildtype (*C57Bl/6J*) controls and were performed by a researcher that was masked to the age and strain of the animals.

To quantify nuclear size and HNA expression, eyes bearing comparable-sized cell masses were used. Nuclear size was determined by selecting cells at random and measuring along the longest axis using ImageJ. Nuclei size in mouse cones were measured using the *Chrnb4.eGFP* cone reporter line. Human nuclei measurements were taken from 2.1L/MOPSIN.GFP^{+ve}/HNA^{+ve} cones within the donor cell. At least N = 3 retinas were used for each group.

Retinal and Visual Function tests

Optomotor response

Ten weeks after human cone transplantation, the optokinetic reflex of recipient mice was assessed using the OptoDrum system (Striatech, Tübingen, Germany). Mice were kept under ambient light conditions and pupil dilation was not performed. Briefly, mice were placed on an elevated central platform surrounded by four computer screens displaying a striped pattern rotating at 12 degree/second. By changing contrast and spatial frequency of the stripes while monitoring the reflexive head movement of the test mouse, the threshold of its vision was determined. The visual acuity was tested at 99.72% contrast and the contrast sensitivity was measured at 0.122 cycle/degree. Note that the maximum irradiance emitted by the screens as measured at the platform was $8.8x10^{13}$ photons cm^2/s. The reduced sensitivity of $Gnat1^{-/-}$ (cone-only) retinas, compared to wildtype mice, indicates that it is unlikely to maximally stimulate human cones. The optokinetic response was automatically detected and analysed by the OptoDrum software in an unbiased manner. The left and right eyes were tested separately by changing the rotating direction of the visual stimulus (clockwise for the left eye and counterclockwise for the right eye). Each eye was

tested on 3 separate occasions within one week. *Gnat1*$^{-/-}$ and *C57Bl/6* wildtype mice served as positive controls for cone-only and rod/cone function. All individual test outcomes from all animals assessed are shown. N = 15 *Aipl1*$^{-/-}$ treated eyes, N = 9 *Aipl1*$^{-/-}$ untreated eyes, N = 6 *Gnat1*$^{-/-}$ eyes and N = 6 *C57Bl/6* eyes were tested. Note that x3 *Aipl1*$^{-/-}$ received no treatment, the remainder had one or both eyes treated.

Electroretinogram recordings (ERGs)

Four *Gnat1*$^{-/-}$, four *Aipl1*$^{-/-}$ hCone transplanted mice and four untreated *Aipl1*$^{-/-}$ mice underwent dark adapted ERG recording using a Celeris ERG system with a full field stimulator (Diagnosys). A masked protocol was employed such that the person performing the ERGs and analysis did not know which eyes received treatment. Animals were dark adapted for a minimum of 6 hours prior to ERG before being anaesthetized as above and pupils dilated using a combination of Tropicamide (1%) and phenylephrine (2.5%). Viscotears were placed on each cornea to keep them moistened and facilitate contact with corneal electrodes. Animals were kept warm throughout recording. The ERG protocol was run in the “Touch/Touch” modality, in which eyes are recorded sequentially with the non-recorded eye acting as the reference electrode. Dark adapted, single flash recordings were obtained at light intensities of 0.001, 0.01, 0.1, 1, 10 and 30 cd.s/m2 with a flash duration of 4ms and interstimulus intervals of 5 s, 5 s, 5 s, 10 s, 30 s, 30 s respectively. Data were recorded from 50 ms before stimulus onset to 300 ms post-stimulus with a sampling frequency of 2 kHz. Ten responses were averaged for light intensities of 0.001, 0.01, 0.1, and 1 cd.s/m2 and 5 responses for 10 and 30 cd.s/m2. The bandpass filter was set between 0.125 and 300 Hz.

For analysis, the a- and b-wave amplitudes (a-wave trough to b-wave peak) were measured. The mean time to peak for *Gnat1*$^{-/-}$ mice (N = 4) was determined for each intensity and this time point used to determine a- and b- wave amplitudes in the other lines, if not clear.

MEA

Gnat1$^{-/-}$ (N = 4), human cone transplanted *Aipl1*$^{-/-}$ (N = 4) and untreated *Aipl1*$^{-/-}$ (N = 3) mice were euthanized by cervical dislocation ~3 months post-transplantation. Eyes were immediately enucleated, and retinal isolation was performed in the dark in warm carboxygenated (95% O2-5% CO2) AMES media supplemented with 1.9 g/L sodium bicarbonate (Sigma Aldrich, UK). The retina was incised four times in a Maltese cross motif and mounted onto a perforated Multi Electrode Array (120pMEA100/30iR-ITO; MultiChannel Systems, Reutlingen, Germany) with the ganglion cell layer facing down onto the electrodes. For transplanted animals, GFP expressing regions of the retinal cell mass were placed centrally over the electrodes to maximize the recording area covered by transplanted human cones. A platinum harp (0.8g) bearing a framework of parallel silicon strings was used to hold the retina in place and keep it stable during recording. The MEA chamber was then mounted into the head stage (MEA2100-120 head stage; Multi Channel Systems). Electrophysiological signals were digitised and recorded with a sampling frequency of 20 kHz using Multi Channel Experimenter (Multi Channel Systems). Prior to recording, the retina was allowed to rest for 30 minutes of dark adaptation and to allow for neuronal activity to stabilize. To preserve physiological conditions, the tissue was perfused with carboxygenated AMES (PPS2; Multi Channel Systems) and 9-cis-retinaldehyde (Sigma Aldrich; UK) and maintained at 36°C (TC02 controller; Multi Channel Systems) throughout the duration of the experiment.

Presentation of visual stimuli

Light stimuli were designed using MC_Stimulus II (Multi Channel systems), which programmed a T-Cube LED Driver (Thorlabs; Germany) to control a mounted Cyan LED (λmax = 505nm; M505L4; Thorlabs). A light guide was used to project light from the LED onto the retina from above (Maximum irradiance at retinal surface = 1.12×10^{15} photons cm^2/s). All light measurements were recorded using a calibrated spectroradiometer (ILT960VIS-RAA4; Pro Lite Technology)

Light stimuli & pharmacology

1s Flashes

Full-field 1 s light steps were presented from darkness with a 10 s interstimulus interval and repeated 10 times.

Sensitivity

In all animals, 10 repeats of a full-field 100 ms light steps with a 10 s interstimulus interval, were presented at nine increasing light intensities from a dark-adapted background (maximum irradiance = 1.12×10^{15} photons cm^2/s). The stimulus sequence started from the lowest light irradiance and ended at the highest.

Pharmacology

The glutamatergic blockers L (+)-2-amino-4-phosphonobutyrate (L-AP4) (group III metabotropic glutamate receptor agonist) (50 µM), 6, 7-dinitroquinoxaline-2, 3-dione (DNQX) (AMPA/kainate receptor antagonist) (40 µM), and d-2-amino-5-phosphonovalerate (d-AP5) (NMDA receptor antagonist; all from Sigma Aldrich, UK) (40 µM) were added to the AMES media, to block synaptic input from outer retinal photoreceptors and identify the origin

of light responses in retinal explants. Under these conditions, we repeated the 1s light pulses described above. Following this protocol, the drugs were washed out and the MEA chamber was cleared with AMES media (Sigma Aldrich, UK) for up to 1 hour before repeating the same 1 s light stimulation protocol.

Data analysis

Spike sorting

Offline, neural waveforms were processed using Offline Sorter (v4.7.1; Plexon). Cross-channel artifacts were identified and removed, and then each channel was analyzed separately. For each channel, single-unit spikes were detected and categorized based on the spike waveform via a principal component analysis, whereby distinct clusters of spikes were readily identifiable and showed a clear refractory period in their interspike interval distribution (>1 ms). Single-unit data were subsequently sent too and stored in NeuroExplorer (v5.437; Nex Technologies, MA) in preparation for further analysis.

Identification of light responses

Spike sorted data in Neuroexplorer files was analyzed by custom written MATLAB codes as reported previously (Ribeiro et al., 2021). The Peri-stimulus time histograms (PSTHs) was calculated with 100 ms bins (over 10 repeated trials). Thresholds were defined using both the amplitude and duration of responses. For increases in firing rate, this was defined as the pre-stimulus baseline +3 standard deviation (SD) with 100 ms duration, while for decreases in firing rate this was the pre-stimulus baseline 2 SD with 300 ms duration. Baseline is defined as the average firing rate in the 2s preceding the stimulus over 10 repeated trials. The rules used to assign each neuronal response to a specified class are described in full in (Ribeiro et al., 2021).

Latency & amplitude analysis

Amplitude and Latencies were calculated for individual light responsive units. Amplitude is defined as the change in firing rate between light onset/offset and peak response. Latency is the time difference between stimulus onset/offset and peak response. Latencies were calculated from smoothed PSTHs with 10ms bin to retain an appropriate time resolution, while amplitude was calculated with a 100ms bin. Latency was then plotted for different component of light-responsive units based upon their classification to the 1s light pulse as above and binned at 10ms. Latency to the ON component of responses (ON, ON Suppressed by Dark and ON-OFF units) was calculated as the time at which maximum firing rate was reached following light onset. Latency to the OFF component of responses (OFF, OFF Suppressed by Light and ON-OFF units) were calculated as the time at which maximum firing rate was reached following light offset.

Sensitivity analysis

Single units were filtered to ensure that the firing rate at the highest irradiance demonstrated a significant change in firing rate that was > 3 SD above the pre-stimulus baseline. If this criterion was met, the response of that unit at the eight lower intensities was used for analysis regardless of whether it crossed the confidence interval. Sensitivity curves were calculated by subtracting the pre-stimulus baseline from the average peak firing rate in response to the 100ms light step

Pharmacology Analysis

Single units were categorised to the 1s light step as defined above. The peak firing rate for each light responsive unit was then calculated under the Pharmacology and Washout

conditions. Peak firing rates were statistically analysed for each unit using a 2-Way ANOVA with Dunnett’s Multiple Comparison Test.

Statistical analysis

All values are presented mean ± SD (standard deviation) unless otherwise stated; N, number of animals, retinas or independent experiments performed, where appropriate; n, number of cells or images examined, where appropriate. For MEA experiments, n = number of single units. Statistical significance was assessed using Graphpad Prism software and denoted as $p<0.05$ = *; $p<0.01$ = **; $p<0.001$ = ***. Appropriate statistical tests were applied including 2 tailed t-test (Mann Whitney), 1-way ANOVA with Tukey’s correction for multiple comparisons, 2-way ANOVA with Bonferroni’s correction, and paired and unpaired T-tests were used to compare latency and amplitude calculations. The test used in each experiment is reported in the Results section.

Figures were generated in Adobe Illustrator, Adobe Photoshop and CorelDraw.

SUPPLEMENTARY TABLES

Antibodies	Source
Rhodospin	Sigma
Mouse Cone Arrestin	Merck Millipore
S-Ospin	Merck Millipore
L/M Opsin	Merck Millipore
Peripherin-2 (PRPH2)	Merck Millipore
PKC-α	Santa Cruz Biotechnology
Calbindin	Swant
Secretagogin (SCGN)	Biovendor
Calretinin	Abcam
GFAP	Calbiochem
Human Cone Arrestin	Novus Biologicals

Human Nuclei	Merck Millipore
CtBP2 (Ribeye)	BD Biosciences
mGluR6	
Peanut agglutinin (PNA) biotinylated	2BScientific, B-1075-5
Donkey anti-mouse Alexa Fluor 488	INVITROGEN, ThermoFisher Scientific
Donkey anti-rabbit Alexa Fluor 405	INVITROGEN, ThermoFisher Scientific
Donkey anti-rabbit Alexa Fluor 546	INVITROGEN, ThermoFisher Scientific
Donkey anti-mouse Alexa Fluor 546	INVITROGEN, ThermoFisher Scientific
Donkey anti-chicken Alexa Fluor 647	INVITROGEN, ThermoFisher Scientific
Donkey anti-rabbit Alexa Fluor 647	INVITROGEN, ThermoFisher Scientific
Streptavidin Alexa Fluor 633 conjugate	INVITROGEN, ThermoFisher Scientific

Supplementary Table 1

Supplementary Table detailing primary and secondary antibodies used.
